# Telomerecat: A ploidy-agnostic method for estimating telomere length from whole genome sequencing data

**DOI:** 10.1038/s41598-017-14403-y

**Published:** 2018-01-22

**Authors:** James H. R. Farmery, Mike L. Smith, NIHR BioResource - Rare Diseases, Aarnoud Huissoon, Abigail Furnell, Adam Mead, Adam P. Levine, Adnan Manzur, Adrian Thrasher, Alan Greenhalgh, Alasdair Parker, Alba Sanchis-Juan, Alex Richter, Alice Gardham, Allan Lawrie, Aman Sohal, Amanda Creaser-Myers, Amy Frary, Andreas Greinacher, Andreas Themistocleous, Andrew J. Peacock, Andrew Marshall, Andrew Mumford, Andrew Rice, Andrew Webster, Angie Brady, Ania Koziell, Ania Manson, Anita Chandra, Anke Hensiek, Anna Huis in’t Veld, Anna Maw, Anne M. Kelly, Anthony Moore, Anton Vonk Noordegraaf, Antony Attwood, Archana Herwadkar, Ardi Ghofrani, Arjan C. Houweling, Barbara Girerd, Bruce Furie, Carmen M. Treacy, Carolyn M. Millar, Carrock Sewell, Catherine Roughley, Catherine Titterton, Catherine Williamson, Charaka Hadinnapola, Charu Deshpande, Cheng-Hock Toh, Chiara Bacchelli, Chris Patch, Chris Van Geet, Christian Babbs, Christine Bryson, Christopher J. Penkett, Christopher J. Rhodes, Christopher Watt, Claire Bethune, Claire Booth, Claire Lentaigne, Coleen McJannet, Colin Church, Courtney French, Crina Samarghitean, Csaba Halmagyi, Daniel Gale, Daniel Greene, Daniel Hart, David Allsup, David Bennett, David Edgar, David G. Kiely, David Gosal, David J. Perry, David Keeling, David Montani, Debbie Shipley, Deborah Whitehorn, Debra Fletcher, Deepa Krishnakumar, Detelina Grozeva, Dinakantha Kumararatne, Dorothy Thompson, Dragana Josifova, Eamonn Maher, Edwin K. S. Wong, Elaine Murphy, Eleanor Dewhurst, Eleni Louka, Elisabeth Rosser, Elizabeth Chalmers, Elizabeth Colby, Elizabeth Drewe, Elizabeth McDermott, Ellen Thomas, Emily Staples, Emma Clement, Emma Matthews, Emma Wakeling, Eric Oksenhendler, Ernest Turro, Evan Reid, Evangeline Wassmer, F. Lucy Raymond, Fengyuan Hu, Fiona Kennedy, Florent Soubrier, Frances Flinter, Gabor Kovacs, Gary Polwarth, Gautum Ambegaonkar, Gavin Arno, Gavin Hudson, Geoff Woods, Gerry Coghlan, Grant Hayman, Gururaj Arumugakani, Gwen Schotte, H. Terry Cook, Hana Alachkar, Hana Lango Allen, Hana Lango-Allen, Hannah Stark, Hans Stauss, Harald Schulze, Harm J. Boggard, Helen Baxendale, Helen Dolling, Helen Firth, Henning Gall, Henry Watson, Hilary Longhurst, Hugh S. Markus, Hugh Watkins, Ilenia Simeoni, Ingrid Emmerson, Irene Roberts, Isabella Quinti, Ivy Wanjiku, J. Simon R. Gibbs, James Thaventhiran, James Whitworth, Jane Hurst, Janine Collins, Jay Suntharalingam, Jeanette Payne, Jecko Thachil, Jennifer M. Martin, Jennifer Martin, Jenny Carmichael, Jesmeen Maimaris, Joan Paterson, Joanna Pepke-Zaba, Johan W. M. Heemskerk, Johanna Gebhart, John Davis, John Pasi, John R. Bradley, John Wharton, Jonathan Stephens, Julia Rankin, Julie Anderson, Julie Vogt, Julie von Ziegenweldt, Karola Rehnstrom, Karyn Megy, Kate Talks, Kathelijne Peerlinck, Katherine Yates, Kathleen Freson, Kathleen Stirrups, Keith Gomez, Kenneth G. C. Smith, Keren Carss, Kevin Rue-Albrecht, Kimberley Gilmour, Larahmie Masati, Laura Scelsi, Laura Southgate, Lavanya Ranganathan, Lionel Ginsberg, Lisa Devlin, Lisa Willcocks, Liz Ormondroyd, Lorena Lorenzo, Lorraine Harper, Louise Allen, Louise Daugherty, Manali Chitre, Manju Kurian, Marc Humbert, Marc Tischkowitz, Maria Bitner-Glindzicz, Marie Erwood, Marie Scully, Marijke Veltman, Mark Caulfield, Mark Layton, Mark McCarthy, Mark Ponsford, Mark Toshner, Marta Bleda, Martin Wilkins, Mary Mathias, Mary Reilly, Maryam Afzal, Matthew Brown, Matthew Rondina, Matthew Stubbs, Matthias Haimel, Melissa Lees, Michael A. Laffan, Michael Browning, Michael Gattens, Michael Richards, Michel Michaelides, Michele P. Lambert, Mike Makris, Minka De Vries, Mohamed Mahdi-Rogers, Moin Saleem, Moira Thomas, Muriel Holder, Mélanie Eyries, Naomi Clements-Brod, Natalie Canham, Natalie Dormand, Natalie Van Zuydam, Nathalie Kingston, Neeti Ghali, Nichola Cooper, Nicholas W. Morrell, Nigel Yeatman, Noémi Roy, Olga Shamardina, Omid S. Alavijeh, Paolo Gresele, Paquita Nurden, Patrick Chinnery, Patrick Deegan, Patrick Yong, Patrick Yu-Wai-Man, Paul A. Corris, Paul Calleja, Paul Gissen, Paula Bolton-Maggs, Paula Rayner-Matthews, Pavandeep K. Ghataorhe, Pavel Gordins, Penelope Stein, Peter Collins, Peter Dixon, Peter Kelleher, Phil Ancliff, Ping Yu, R. Campbell Tait, Rachel Linger, Rainer Doffinger, Rajiv Machado, Rashid Kazmi, Ravishankar Sargur, Remi Favier, Rhea Tan, Ri Liesner, Richard Antrobus, Richard Sandford, Richard Scott, Richard Trembath, Rita Horvath, Rob Hadden, Rob V. MackenzieRoss, Robert Henderson, Robert MacLaren, Roger James, Rohit Ghurye, Rosa DaCosta, Rosie Hague, Rutendo Mapeta, Ruth Armstrong, Sadia Noorani, Sai Murng, Saikat Santra, Salih Tuna, Sally Johnson, Sam Chong, Sara Lear, Sara Walker, Sarah Goddard, Sarah Mangles, Sarah Westbury, Sarju Mehta, Scott Hackett, Sergey Nejentsev, Shahin Moledina, Shahnaz Bibi, Sharon Meehan, Shokri Othman, Shoshana Revel-Vilk, Simon Holden, Simon McGowan, Simon Staines, Sinisa Savic, Siobhan Burns, Sofia Grigoriadou, Sofia Papadia, Sofie Ashford, Sol Schulman, Sonia Ali, Soo-Mi Park, Sophie Davies, Sophie Stock, Souad Ali, Sri V. V. Deevi, Stefan Gräf, Stefano Ghio, Stephen J. Wort, Stephen Jolles, Steve Austin, Steve Welch, Stuart Meacham, Stuart Rankin, Suellen Walker, Suranjith Seneviratne, Susan Holder, Suthesh Sivapalaratnam, Sylvia Richardson, Taco Kuijpers, Taco W. Kuijpers, Tadbir K. Bariana, Tamam Bakchoul, Tamara Everington, Tara Renton, Tim Young, Timothy Aitman, Timothy Q. Warner, Tom Vale, Tracey Hammerton, Val Pollock, Vera Matser, Victoria Cookson, Virginia Clowes, Waseem Qasim, Wei Wei, Wendy N. Erber, Willem H. Ouwehand, William Astle, William Egner, Wojciech Turek, Yvonne Henskens, Yvonne Tan, Andy G. Lynch

**Affiliations:** 1 Cancer Research UK Cambridge Institute, University of Cambridge, Li Ka Shing Centre, Robinson Way, Cambridge, CB2 0RE UK; 2 European Molecular Biology Laboratory (EMBL), Genome Biology Unit, 69117 Heidelberg, Germany; 3 School of Mathematics and Statistics/School of Medicine, University of St Andrews, St Andrews, Fife, KY16 9SS UK; 4 Birmingham Heartlands, Bordesley Green E, Birmingham, B9 5SS UK; 5 University of Cambridge, The Old Schools, Trinity Lane, Cambridge, CB2 1TN UK; 6 Oxford University Hospitals NHS Foundation Trust, John Radcliffe Hospital, Headley Way, Headington, Oxford, OX3 9DU UK; 7 University of Oxford, University Offices, Wellington Square, Oxford, OX1 2JD UK; 8 Centre for Nephrology, University College London, UCL Medical School, Rowland Hill Street, London, NW3 2PF UK; 9 Great Ormond Street Hospital for Children NHS Foundation Trust, Great Ormond Street, London, WC1N 3JH UK; 10 UCL Great Ormond Street Institute of Child Health, 30 Guilford St, London, WC1N 1EH UK; 11 Newcastle Freeman Hospital, Freeman Rd, High Heaton Newcastle upon Tyne, NE7 7DN UK; 12 Cambridge University Hospitals NHS Foundation Trust, Addenbrookes Hospital, Hills Rd, Cambridge, CB2 0QQ UK; 13 University Hospitals Birmingham, Mindelsohn Way, Edgbaston Birmingham, B15 2TH UK; 14 Sheffield CRF, Royal Hallamshire, Royal Hallamshire Hospital, Glossop Road, Sheffield, S10 2JF UK; 15 Birmingham Children’s Hospital NHS Foundation Trust, Steelhouse Ln, Birmingham, B4 6NH UK; 16 Institute for Immunology and Transfusion Medicine, Ernst-Moritz-Arndt-University of Greifswald, Domstraße 11, 17489 Greifswald, Germany; 17 Golden Jubilee National Hospital, Agamemnon St, Clydebank, G81 4DY UK; 18 Salford Royal NHS Foundation Trust, Stott Ln, Salford, M6 8HD UK; 19 University Hospitals Bristol NHS Foundation Trust, Trust Headquarters, Marlborough Street, Bristol, BS1 3NU UK; 20 University of Bristol, Senate House, Tyndall Avenue, Bristol, BS8 1TH UK; 21 Imperial College, Kensington London, SW7 2AZ UK; 22 Moorfields Eye Hospital NHS Foundation Trust, 162 City Road, London, EC1V 2PD UK; 23 University College London, Gower St, Bloomsbury, London, WC1E 6BT UK; 24 London North West Healthcare NHS Trust, Northwick Park Hospital, Watford Road, Harrow, HA1 3UJ UK; 25 Guy’s and St Thomas’ NHS Foundation Trust, St Thomas’ Hospital, Westminster Bridge Road, London, SE1 7EH UK; 26 VU University Medical Center, De Boelelaan, 1117, 1081 HV Amsterdam, Netherlands; 27 University of Giessen, Ludwigstraße 23, 35390 Gießen, Germany; 28 University of South Paris, 15 Rue Georges Clemenceau, 91400 Orsay, France; 29 Beth Israel Deaconess Medical Centre, Harvard Medical School, 330 Brookline Ave, Boston, MA 02215 USA; 30 Department of Medicine, University of Cambridge, Addenbrooke’s Hospital, Hills Rd, Cambridge, CB2 0SP UK; 31 Imperial College Healthcare NHS Trust, The Bays, St Mary’s Hospital, South Wharf Road, London, W2 1NY UK; 32 Scunthorpe General Hospital, Cliff Gardens, Scunthorpe, DN15 7BH UK; 33 Haemophilia Centre, Kent & Canterbury Hospital, East Kent Hospitals University Foundation Trust, Ethelbert Road, Canterbury, Kent TN24 OLZ UK; 34 King’s College, Strand, London, WC2R 2LS UK; 35 The Roald Dahl Haemophilia Centre, Royal Liverpool Hospital, Prescot St, Liverpool, L7 8XP UK; 36 Department of Cardiovascular Sciences, Center for Molecular and Vascular Biology, University of Leuven, Oude Markt 13, 3000 Leuven, Belgium; 37 Imperial and Hammersmith Hospitals, Du Cane Rd, Shepherd’s Bush, London, W12 0HS UK; 38 Plymouth Hopsital, Derriford Road, Crownhill, Clymouth, Devon PL6 8DH UK; 39 Department of Haematology, University of Cambridge, Wellcome Trust Mrc Bldg, Addenbrookes Hospital, Hills Rd, Cambridge, CB2 0XY UK; 40 MRC-BSU, Cambridge Institute of Public Health, Forvie Site, Robinson Way, Cambridge Biomedical Campus, Cambridge, CB2 0SR UK; 41 The Royal London Hospital, Barts Health NHS Trust, Whitechapel Rd, Whitechapel, E1 1BB UK; 42 Department of Haematology, Castle Hill Hospital, Hull and East Yorkshire NHS Foundation Trust, Castle Road, Cottingham, HU16 5JQ UK; 43 Royal Hospitals Belfast, Trust Headquarters, A Floor, Belfast City Hospital, Lisburn Road, Belfast, BT9 7AB UK; 44 Oxford Haemophilia and Thrombosis Centre, Oxford University Hospitals NHS Trust, The Churchill Hospital, Churchill Hospital, Oxford, OX3 7LE UK; 45 University of Cambridge (CIMR Medical Genetics), Cambridge Institute for Medical Research, University of Cambridge, Cambridge Biomedical Campus, Wellcome Trust/MRC Building, Hills Road, Cambridge, CB2 0XY UK; 46 National Renal Complement Therapeutics Centre, Newcastle University, Royal Victoria Infirmary - Victoria Wing, Newcastle upon Tyne, NE1 4LP UK; 47 University College London Hospitals NHS Foundation Trust, 235 Euston Rd, Bloomsbury London, NW1 2BU UK; 48 Royal Hospital for Children, NHS Greater Glasgow and Clyde, 1345 Govan Rd, Glasgow, G51 4TF UK; 49 Nottingham University Hospitals NHS Trust, Hucknall Rd, Nottingham, NG5 1PB UK; 50 The National Hospitals for Neurology and Neurosurgery, UCLH and UCL, National Hospital for Neurology & Neurosurgery, Queen Square London, WC1N 3BG UK; 51 Hopital St Louis, 1 Avenue Claude Vellefaux, 75010 Paris, France; 52 University of Sorbonne, 75005 Paris, France; 53 University of Graz, 8010, Universit ätspl. 3, 8010 Graz, Austria; 54 Papworth Hospital, Papworth Everard, Cambridge, CB23 3RE UK; 55 Royal Free Hospital, Pond St, Hampstead London, NW3 2cvG UK; 56 Epsom & St Helier University Hospitals NHS Trust, Wrythe Ln, Sutton Carshalton, SM5 1AA UK; 57 Leeds Teaching Hospitals NHS Foundation Trust, Great George Street, Leeds, West Yorkshire LS1 3EX UK; 58 Centre for Complement and Inflammation Research, Imperial College, London, SW7 2AZ UK; 59 Lehrstuhl für Experimentelle Biomedizin, Universitätsklinikum Würzburg, Josef-Schneider-Straße 2, 97080 Würzburg, Germany; 60 Aberdeen Royal Infirmary, Foresterhill, Aberdeen, AB25 2ZN UK; 61 Barts Health NHS Trust, Turner St, Whitechapel London, E1 1BB UK; 62 Newcastle University, Newcastle upon Tyne, NE1 7RU UK; 63 Sapienza Universita di Roma, Piazzale Aldo Moro, 5, 00185 Roma, RM Italy; 64 National Heart & Lung Institute, Imperial College, Dovehouse Street, London, SW3 6LR UK; 65 Royal United Bath Hospitals, Combe Park, Avon, BA1 3NG UK; 66 Department of Haematology, Sheffield Children’s Hospital NHS Foundation Trust, Western Bank Sheffield, S10 2TH UK; 67 Haematology Department, Manchester Royal Infirmary, Oxford Rd, Manchester, M13 9WL UK; 68 Maastricht University, Minderbroedersberg 4-6, 6211 LKZ Maastricht, Netherlands; 69 Medical University of Vienna, Spitalgasse 23, 1090 Wien, Austria; 70 Royal Devon & Exeter NHS Foundation Trust, Barrack Road, Exeter, Devon EX2 5DW UK; 71 Haematology Department, Royal Victoria Infirmary, Queen Victoria Rd, Newcastle upon Tyne, NE1 4LP UK; 72 The Katharine Dormandy Haemophilia Centre and Thrombosis Unit, Royal Free London NHS Foundation Trust, Pond St, Hampstead London, NW3 2QG UK; 73 San Matteo, Pavia, Viale Camillo Golgi, 19, 27100 Pavia, PV Italy; 74 Birmingham University NHS Foundation Trust, Level 1, Queen Elizabeth Hospital Birmingham, Mindelsohn Way, Edgbaston Birmingham, B15 2GW UK; 75 Queen Mary University of London, Mile End Rd, London, E1 4CS UK; 76 University Hospital Wales, Cardiff and Vale UHB Headquarters, University Hospital of Wales (UHW), Heath Park, Cardiff, CF14 4XW UK; 77 Department of Haematology, Great Ormond Street Hospital for Children NHS Trust, Great Ormond Street, London, WC1N 3JH UK; 78 Madsen Health Center, 555 Foothill Dr, Salt Lake City, UT 84112 USA; 79 Leicester Royal Infirmary, Infirmary Square, Leicester, LE1 5WW UK; 80 Department of Pediatrics, Perelman School of Medicine at the University of Pennsylvania, 34th Street & Civic Center Boulevard, Philadelphia, PA 19104 USA; 81 Division of Hematology, Children’s Hospital of Philadelphia, 3401 Civic Center Blvd, Philadelphia, PA 19104 USA; 82 Royal Hallamshire NHS Foundation Trust, Glossop Road, Sheffield, S10 2JF UK; 83 Maastricht University Medical Centre, Postbus, 5800, 6202 AZ Maastricht, Netherlands; 84 King’s College Hospital NHS foundation trust, Denmark Hill, Brixton London, SE5 9RS UK; 85 Gartnavel General Hospital, NHS Greater Glasgow and Clyde, 1055 Great Western Rd, Glasgow, G12 0XH UK; 86 Royal Brompton Hospital, Sydney St, Chelsea London, SW3 6NP UK; 87 University of Perugia, Piazza dell’Università, 06123 Perugia, PG Italy; 88 Institut Hospitalo-Universitaire LIRYC, PTIB, Hopital Xavier Arnozan, Pessac, Avenue du Haut Lévêque, 33604 Pessac, France; 89 Frimley Park Hospital, Portsmouth Rd, Frimley Camberley, GU16 7UJ UK; 90 NHS Blood and Transplant, Manchester Blood Centre, Plymouth Grove Manchester, M13 9LL UK; 91 Hull & East Yorkshire Hospitals NHS Trust, Anlaby Rd, Hull, HU3 2JZ UK; 92 Arthur Bloom Haemophilia Centre, University Hospital of Wales Heath Park, Cardiff, Wales, Heath Park Way Cardiff, CF14 4XW UK; 93 Glasgow Royal Infirmary, NHS Greater Glasgow and Clyde, 84 Castle St, Glasgow, G4 0SF UK; 94 University of Lincoln, Brayford Pool, Lincoln, LN6 7TS UK; 95 Southampton General Hospital, University Hospital Southampton NHS Foundation Trust, Tremona Road, Southampton Hampshire, SO16 6YD UK; 96 Sheffield Teaching Hospitals, Herries Road, Sheffield, S5 7AU UK; 97 Haematological Laboratory, Trousseau Children’s Hospital, 26 Avenue du Dr Arnold Netter, 75012 Paris, France; 98 Sandwell and West Birmingham Hospitals, Dudley Road, Birmingham, West Midlands B18 7QH UK; 99 Norfolk & Norwich University Hospital, Colney Ln, Norwich, NR4 7UY UK; 100 University Hospitals of North Midlands, Royal Stoke University Hospital, Newcastle Road, Stoke-on-Trent, ST4 6QG UK; 101 Haemophilia, Haemostasis and Thrombosis Centre, Hampshire Hospitals NHS Foundation Trust, Aldermaston Rd, Basingstoke, RG24 9NA UK; 102 Hadassah-Hebrew University Hospital, Jerusalem, 91120 Israel; 103 Department of Haematology, Guys and St Thomas’ NHS Foundation Trust, Guy’s Hospital, Great Maze Pond, London, SE1 9RT UK; 104 Emma Children’s Hospital AMC, Meibergdreef 9, 1105 AZ Amsterdam-Zuidoost, Netherlands; 105 Salisbury Hospital, Salisbury NHS Foundation Trust, Odstock Rd, Salisbury, SP2 8BJ UK; 106 University of Edinburgh, Old College, South Bridge, Edinburgh, EH8 9YL UK; 107 Pathology and Laboratory Medicine, University of Western Australia, Crawley, Western Australia, 35 Stirling Hwy, Crawley, WA 6009 Australia; 108 Wellcome Trust Sanger Institute, Wellcome Trust Genome Campus, Hinxton, CB10 1SA UK

## Abstract

Telomere length is a risk factor in disease and the dynamics of telomere length are crucial to our understanding of cell replication and vitality. The proliferation of whole genome sequencing represents an unprecedented opportunity to glean new insights into telomere biology on a previously unimaginable scale. To this end, a number of approaches for estimating telomere length from whole-genome sequencing data have been proposed. Here we present Telomerecat, a novel approach to the estimation of telomere length. Previous methods have been dependent on the number of telomeres present in a cell being known, which may be problematic when analysing aneuploid cancer data and non-human samples. Telomerecat is designed to be agnostic to the number of telomeres present, making it suited for the purpose of estimating telomere length in cancer studies. Telomerecat also accounts for interstitial telomeric reads and presents a novel approach to dealing with sequencing errors. We show that Telomerecat performs well at telomere length estimation when compared to leading experimental and computational methods. Furthermore, we show that it detects expected patterns in longitudinal data, repeated measurements, and cross-species comparisons. We also apply the method to a cancer cell data, uncovering an interesting relationship with the underlying telomerase genotype.

## Introduction

Telomeres are the ribonucleoprotein structures that shield the ends of chromosomes from DNA damage responses^1^. They are multifunctional regions of the genome that, unless being actively lengthened (by e.g. telomerase) will shorten with DNA duplication^2^. In this manner they both act as a molecular clock and provide a natural limit on the replicative potential of a cell, with possible pathways to apoptosis, senescence and, in cancer cells, genomic instability^3^. Telomere length is thus not only a risk factor for cancer and other diseases^4^, with germline mutations near to TERT (the gene encoding telomerase) being associated with several cancers^5^, but also has a mechanistic role in tumour aetiology through driving instability, influencing regulation of telomere-proximal genes^6^, and (through activation of telomere-lengthening) provision of replicative immortality^7^. In humans, the DNA component of telomere is an extremely repetitive region of the genome comprised of the nucleotide hexamer: (*TTAGGG*)_*n*_.

In this study we present Telomerecat, the first tool designed specifically to estimate mean telomere length from cancer whole genome sequencing (WGS) data. There have been previous approaches to using WGS data to say something about telomeres. Castle *et al*. provided a proof of concept in 2010^8^, and this was refined by the first group to use such an approach in earnest^9^. Ding *et al*.^10^ published the first fully-fledged method for estimating length rather than just telomere content, with the accompanying tool ‘TelSeq’. Their study was also the first time a computational method had been validated against an established experimental method.

TelSeq assumes a fixed number of chromosomes when estimating telomere length and so makes no allowance for aneuploidy. Nevertheless, as the strongest available tool there are several examples of TelSeq being used to analyse cancer datasets^11,12^. Ṅotably a recent pan-cancer analysis made use of the TelSeq tool^6^. While generally sound, such analyses are vulnerable to misinterpretation in the event of systematic differences in aneuploidy (as may be the case when comparing different cancer types). Indeed, recurrent somatic copy number alterations involving the telomere were observed in all cancer types studied in a pan-cancer study of Cancer Genome Atlas data^13^.

Where such changes (suggestive of aneuploidy) occur, cells will likely be left with an altered number of telomeres. Accordingly the quantity (and proportion) of telomere sequence within the sample is altered, even if the mean length of telomeres is unaltered. Thus if we observe more telomere sequence in a cancer sample, we do not know if this is due to longer telomeres.

Two other tools of note have been published: Telomere Hunter and Computel. TelomereHunter^14^ reports telomere content rather than telomere length, and so does not provide a direct comparison. TelomereHunter classifies reads based on their mapping location within the parent BAM file and outputs statistics relating to variations of the canonic telomere hexamer. Computel^15^ does allow the user to specify the number of telomeres present, but since this is unknown (and cannot safely be inferred from copy-number profiles or ploidy statistics) it again does not provide a direct comparison. Since TelSeq is more frequently used in the literature, has greater experimental validation than Computel, and a recent comparison study^16^ did not find that the greater convenience of TelSeq was at the cost of poorer performance, we take TelSeq as the representative of current methods in our comparisons.

Rather than normalizing against the entire genome, Telomerecat normalizes the telomeric content against the subtelomeric regions. In this manner it is agnostic to the ploidy of the sample, and assumes only that each telomere has a subtelomere.

Erroneous regions of apparent telomere and subtelomere can arise from other stretches of the TTAGGG repeat sequence that appear in the human genome: so-called Interstitial telomeric repeats (‘ITRs’)^17^. Telomerecat estimates and corrects for the number of ITR-originating reads by assuming that the aggregate number of reads from the 3′ end of TTAGGG ITR sequences will be approximately equal to the aggregate number of reads from the 5′ end, while true telomeres only have a boundary at one end. In this manner, telomerecat obtains an estimate of ITR contributions without having to align to these difficult-to-map regions.

A third potential hindrance for telomere estimation, after aneuploidy and ITRs, is that it is difficult to define the end of the telomere precisely, based solely on genomic sequence (explicit information about DNA secondary structures and the locations of bound proteins having been lost). The subtelomere is composed of subtelomeric repeat sequences and segmental duplicates, interspersed by canonic telomere repeats^18^. These subtelomeric repeat sequences can look much like the telomere but with the addition of sequencing errors. Too strict a definition of telomere as being the region of TTAGGG repeats would be hostage to genuine variations, sequencing errors, and somatic mutations.

Telomere length is therefore necessarily a subjective measure, consistent only within the method used. Accordingly there may be an off-set in comparisons with other methods. Even ‘gold standard’ laboratory methods for measuring telomere lengths may have their own biases in this regard^19^.

Core to Telomerecat’s estimation process is the ratio between read-pairs that lie within the telomere and read-pairs that span the telomere boundary. Observing reads on the boundary between telomere and subtelomere provides a quantification of telomere numbers through which we normalize the telomere lengths. Where other samples always assume that more telomere reads mean longer telomere, Telomerecat is able to account for the fact that there may actually be more individual telomeres.

Moreover, differences in patterns of sequencing error have the potential to lead to inconsistency between samples even if using the same method. To this end, Telomerecat includes a novel method for correcting sequencing error in telomere sequencing reads. This model automatically adapts to differing error across sequencing preparations.

Telomerecat is an open source tool, the code is available from https://github.com/jhrf/telomerecat. Full installation and usage documentation is available at https://telomerecat.readthedocs.io.

## Results

### Validation in presumed-diploid blood samples

**Figure 1:**
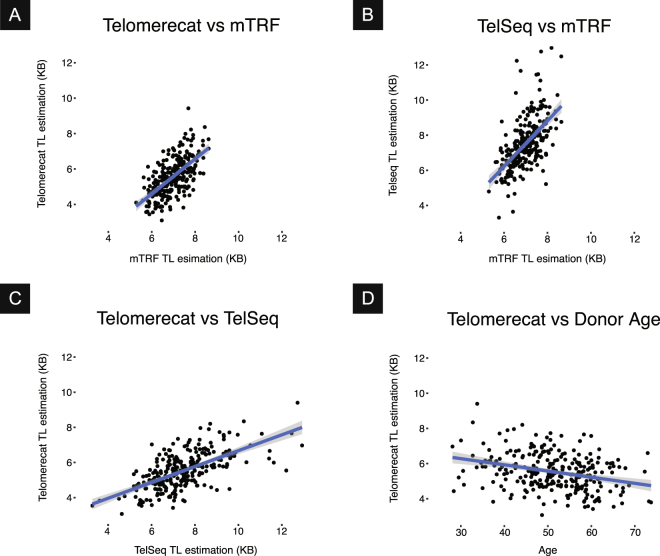
Scatter plots describing the relationship between Telomerecat, mTRF, and TelSeq estimates of telomere length (TL).

To verify that Telomerecat is able to identify telomere length within WGS samples, we compared the algorithm to an established experimental method (mean terminal restriction fragment Southern blot experiment (mTRF)) and the current leading computational method (TelSeq). Blood samples were taken from 260 adult females as part of the TwinsUK10K study, WGS and mTRF were conducted on each sample (described previously^20,21^). The donor’s age at sample collection is also recorded for each sample. Since absolute agreement is not expected, we consider correlations between the methods. The results of the comparisons are shown in Table 1 and in Fig. 1.

**Table 1:** Results for the comparisons between Telomerecat, TelSeq, mTRF and Donor Age.

	Telomerecat	TelSeq	mTRF
TelSeq	*ρ* = 0.631	—	—
mTRF	*ρ* = 0.618	*ρ* = 0.583	—
Donor Age	*ρ* = −0.306	*ρ* = −0.239	*ρ* = −0.321

We observe that the best correlation is between the the two computational methods at $\rho =0.631$. The next best correlation was between mTRF and Telomerecat indicating that Telomerecat agrees with the established experimental method. Both Telomerecat and TelSeq correlate well with mTRF indicating that both tools are providing realistic estimates of telomere length. The extent that Telomerecat correlates with mTRF is in line with correlations previously observed between other experimental methods and mTRF^19^.

Telomerecat estimates telomere length that is shorter, on average, than TelSeq. At least part of this disparity may be due to Telomerecat’s active filtering of reads from ITRs. Telomerecat finds that, on average 7% of telomeric read-pairs identified are from ITRs.

Telomerecat was able to identify a correlation with age only slightly weaker than that of mTRF, a strong indicator that we are capturing genuine information about telomere lengths.

### Application to a longitudinal MSC data set

We applied Telomerecat to a set of WGS samples from a mesenchymal stem cell (MSC) experiment described previously^22^. Mesenchymal stem cells are multipotent stromal cells commonly located in bone marrow^23^. The experiment constituted six WGS samples: an *in vivo* MSC sample from a healthy 31 year old male, three passaged MSC samples (P1,P8 and P13) and two induced pluripotent stem cell (iPSC) samples.

MSCs are unusual amongst mature human stem cells as they do not express any measurable amount of telomerase^24^. Accordingly, telomere length attrition has been described in MSC passage experiments^25,26^. Conversely, iPSC cells have been shown to exhibit heightened telomerase expression^27^. We hypothesised that telomere length would shorten across the passaged MSC samples and lengthen within the iPSC samples.

**Figure 2:**
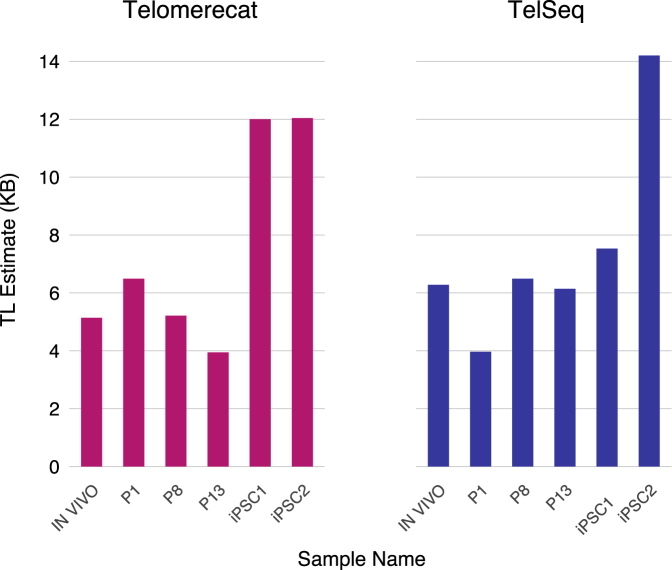
This figure shows estimates for the MSC samples produced by Telomerecat (left) and TelSeq (right). We expect to see a decrease in telomere length with additional passaging (P1 to P13), but consistent high telomere lengths in the two iPSC samples (iPSC1 and iPSC2).

The results of applying Telomerecat and TelSeq to the aforementioned MSC WGS data are shown in Fig. 2. Telomerecat identifies telomere shortening across the passaged samples, as expected. Telomerecat estimates that between P1 and P13 the average telomere length was shortened by 2.5 KB, at a rate of approximately 0.2KB per passage. Furthermore, we see that Telomerecat identifies long telomere length in the the two iPSC samples. We also note that TelSeq fails to identify the expected telomere dynamics. Possible explanations for this discrepancy are discussed in detail in the Supplementary Information Section 2.

### Application to a cancer dataset

After establishing that Telomerecat performs well in diploid samples, we demonstrated that it can also be applied to cancer samples. We applied Telomerecat to a data set comprised of samples from four donors suffering from Hepatocellular carcinoma (HCC)^28^. Primary HCC cells were extracted from each donor in that study. These primary cells were cultured to create cell lines. Samples of the primary cells *in vitro*, an early passage and a late passage were taken for sequencing. Table 2 lists the exact passage number for each sample.

**Table 2:** Patients in the HCC study.

	CLC11	CLC13	CLC16	CLC5
Early Passage Count	6	3	3	7
Late Passage Count	24	18	21	27
TERT Promoter Mutation	No	Yes	Yes	Yes
TERT Amplification	Yes	Yes	No	No
HBV Integration	Yes	No	No	No

**Figure 3:**
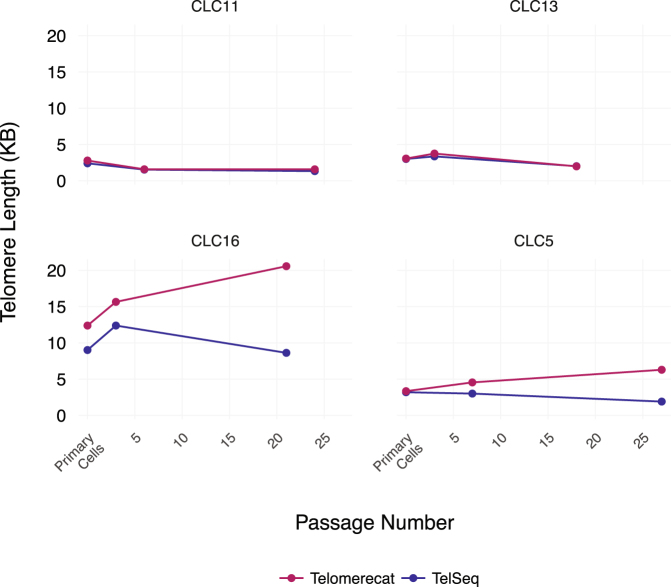
Telomerecat and TelSeq estimates for the HCC cell line dataset.

Figure 3 shows the results of applying Telomerecat and TelSeq to the HCC cohort.

Telomerecat and TelSeq agree on CLC11 and CLC13 with both tools reporting only slight changes in telomere length across the passage experiment. However, the tools seem to diverge in their estimations for CLC16 and CLC5.

Telomerecat identified two telomere length phenotypes across the four donors. CLC11 and CLC13 show a telomere length that is not altered across the passage process. By contrast, in CLC16 and CLC5 we see that telomere length increases across the passaged samples. Z. Qiu *et. al* report that all four samples contain corruptions in the TERT gene as shown in Table 2. It is interesting to note that CLC16 and CLC5 share both a TERT genotype and telomere length phenotype. Previous studies suggest that the presence of TERT promoter mutations and HBV Integration increases TERT expression^29,30^. However it is not clear that heightened expression is indicative of longer telomere lengths. Indeed, HCC tumours generally have shorter telomeres than adjacent normal cells^31^.

Although suggestive, further study and experimentation is required to ascertain the true nature between the underlying genotype and telomere length phenotype amongst cases of HCC.

### Application to a set of repeated measurements

We have also tested Telomerecat on pairs of WGS repeated measurements from the NIHR BioResource - Rare Diseases study. Telomerecat was applied to 93 samples of DNA extracted from whole blood. For each participant two samples were taken. Each sample was sequenced on either the HiSeq. 2000 or HiSeqX platform. We observe cases in this cohort where samples from the same participant were sequenced on the same technology and where samples were sequenced on different technologies. The blood samples from donor pairs were taken on separate occasions up to 3 years apart.

**Figure 4:**
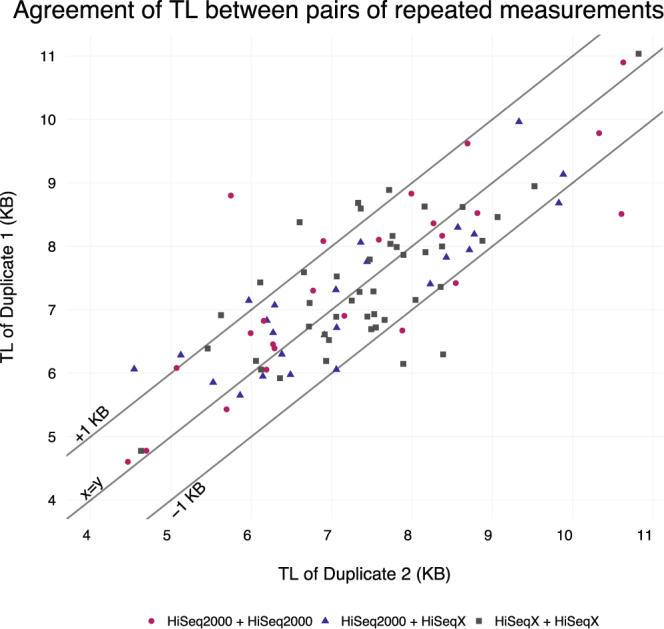
A plot of telomere length (TL) estimates for repeated measurement pairs. Colours correspond to the sequencing platform of each sample in the pair.

A sound approach to telomere-length estimation will be reproducible across duplicate samples. After accounting for batch effects relating to choice of platform, Telomerecat achieves good agreement between the repeat measurements, as shown in Fig. 4.

We observe that estimates from the two measurements show a Pearson correlation of *r* = 0.8. We see that in 80% of the duplicate pairs the difference in estimation is less than 1KB. Previous work suggests that the mTRF has a resolution of 1KB (although other methods have higher resolution)^32^. The fact that Telomerecat identifies displays a similar accuracy on a set of repeat measurements is a reassuring sign, especially given that we expect a certain amount of technical noise and true biological difference between the telomere length of these biological duplicates.

### Application to mouse samples

Mouse telomeres are known to be longer than human telomeres^33^. However, telomere length is known to vary across different mouse strains. We applied Telomerecat to 10 samples from the Mouse Genomes Project^34^.

**Figure 5:**
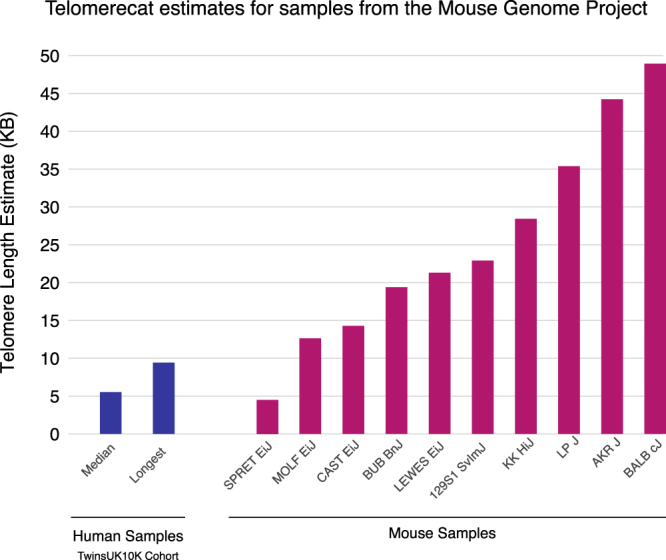
Telomere length estimates by Telomerecat for 10 mouse samples from the Mouse Genomes Project.

Telomerecat identifies a range of telomere lengths, most of which are substantially greater than estimates from human samples. The estimates for the mouse samples, as well as two human samples for comparison, are shown in Fig. 5. TelSeq was not applied as the tool is specifically tailored to the human genome.

Telomerecat identifies a range of telomere lengths for the mice, almost all of the lengths are substantially longer than the longest human telomeres in the TwinsUK10K cohort. Additionally, we note that two of the samples with the shortest estimates - CAST Eij and SPRET Eij - have been identified as having comparatively short telomeres^35–37^. We also note that previous studies have identified the BALB cJ mouse strain as having long telomeres^37^.

### A comparison of running time and resource allocation

Benchmarking was conducted on a MacPro desktop computer with 2 × 2.93 Ghz Quadcore Intel Xeon processors and 16GB of 1066Mhz DDR3 memory. The results of benchmarking for the Telomerecat and TelSeq tools can be found in Table 3. Benchmarking was conducted on QTL190044 from the TwinsUK10K cohort. The results displayed are the average from the three runs.

**Table 3:** Benchmarking results for Telomerecat and TelSeq.

	Telomerecat	TelSeq
Time Taken (seconds)	756	3894
Reads per hour	1.562 × 10^9^	3.299 × 10^8^
Max. Processor Usage (%)	537.6	96.8
Avg. Processor Usage (%)	356.8	80
Max. Memory Usage (GB)	1.9	0.104
Avg. Memory Usage (GB)	1.3	0.037

## Discussion

Here we have demonstrated and validated a novel approach to estimating telomere length from WGS data. Importantly, Telomerecat is the first tool designed to be applicable to cancer experiments as it does not assume a given number of telomeres.

We have validated Telomerecat by showing that it correlates with existing computational and experimental methods as well as with sample donor age. mTRF itself provides an imperfect measure of telomere length and, from correlations with age, it seems that computational methods may be capturing as much information as that approach. Specific wet-lab methods for estimating telomere length will likely remain the gold standard, but given the number of public initiatives generating large sets of sequencing data without matched telomere measurements, improved methods for estimating telomere length from WGS data will always be desirable.

WGS-based methods will naturally become more accurate as the depth of sequencing increases. Much of the inaccuracy in the estimates of the TwinsUK10K data may be attributable to the relatively low coverage of those WGS data. At low coverage, Telomerecat’s estimate of the number of reads crossing the boundary is less certain. As coverage at the boundary decreases and the observed read counts for each individual sample become less certain Telomerecat relies more on the cohort error adjustment (discussed in the methods section). With higher coverage we would expect even better agreement between Telomerecat and the other methods for diploid cells.

We have demonstrated here that Telomerecat is capable of producing estimates that are at least as accurate as computational methods that make an assumption about the number of chromosomes or telomeres, when applied to samples which are presumed to meet this assumption. When the assumption of number of telomeres doesn’t hold, it is reasonable to assume that Telomerecat will still do at least as well, and most likely will do better, as the other methods must see a drop off in accuracy through making such an assumption erroneously. In Section 2 of Supplementary Information we show through simulated data that Telomerecat is not biased by the true chromosome count. There are limited gold standard data available to demonstrate the advantages empirically, but if two well-matched methods differ in their estimates for a particular case, and the first makes an assumption that for that case is demonstrably wrong, it is logical to give credence to the second.

By applying Telomerecat to the duplicate blood samples we have demonstrated Telomerecat’s ability to generate meaningful results on two of the most popular Illumina paired-end platforms. As well as confirming the reliability of Telomerecat’s telomere length estimates, this shows that the estimates are robust to sequencing batches once batch effects are accounted for.

Amongst the most striking results presented here is the estimation of telomere length across MSC cell line passaged data. Telomerecat identifies a clear deterioration of telomere length across the passaged cells and an increase of telomere length in the iPSC samples, in which telomerase had been reactivated. TelSeq fails to identify this pattern.

We see that the most likely reason for TelSeq’s failure to observe the expected telomere dynamics is in the GC correction part of the algorithm (see Section 2 of Supplementary Information for more detailed analysis). This indicates that the relationship between coverage at locations where genomic GC is identical to telomere and actual telomere, on which TelSeq relies, may not always be consistent across experiments.

We have presented the first application of a WGS telomere length estimation approach to data derived from non human samples; Telomerecat’s agnosticism to telomere numbers provides a natural advantage here also. As expected, Telomerecat identifies long telomere length in most of the mice samples. Pleasingly, Telomerecat is concordant with the literature in demonstrating the short telomeres in CAST EiJ and SPRET cJ samples and long telomeres in BALB cJ.

Telomerecat tends to report shorter telomere length than other methods, both computational and experimental. There will be several contributing factors, including disagreement over the definition of the telomere/sub-telomere boundary, and the stringency for categorizing read-pairs as being telomeric. One clear contributing factor in the comparison of computational methods will be Telomerecat’s exclusion of ITR read-pairs, typically contributing 4% to 10% of apparently telomeric read-pairs.

We have also demonstrated that Telomerecat can be run quickly (five times faster than TelSeq for our example). Telomerecat is able to process samples quickly as it is built on a parallel BAM processing framework - parabam^38^ - and thus uses multiple processing cores at all stages of the analysis. Telomerecat promotes reproducible research by generating subsets of reads from which telomere length estimates can be generated. We hope that these smaller file will be more easily stored and transferred allowing researchers to regenerate estimates without the need to process the cumbersome original BAM files.

Finally, we have demonstrated the application to a cancer WGS dataset: Telomerecat’s raison d’être. We see that Telomerecat identifies differing telomere phenotypes across four passage experiments. Intriguingly the two experiments with the most similar telomere length phenotype have an identical underlying TERT corruption.

## Methods

### Overview

**Figure 6:**
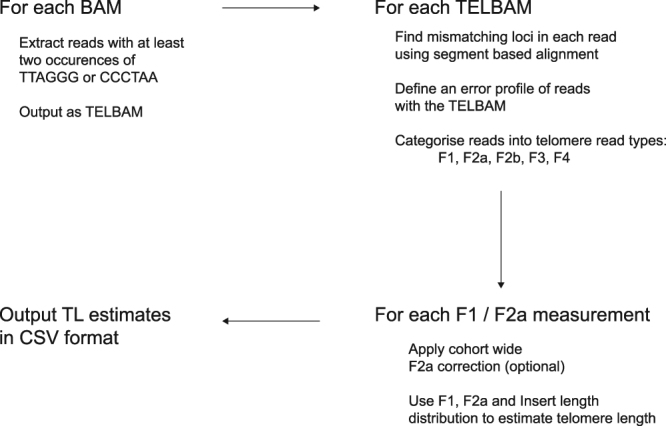
An overview of the Telomerecat length estimation process.

Telomerecat functions as three discrete operations: TELBAM generation, read categorisation and length estimation. A flowchart depicting the method is given in Fig. 6.

First, we collect a relevant subset of reads and their pairs from a BAM file. This subset is referred to as a TELBAM and consists of read pairs where one end of a read pair has two occurrences of the telomeric hexamer. This read subsetting operation is expedited by using the parallel processing framework parabam^38^. We observe that TELBAMs contain less than one ten-thousandth of the reads from an input BAM file.

Next we categorise read pairs according to their sequence composition and orientation on the genome. The telomere length estimate is informed by a ratio of complete telomere read pairs to read pairs on the boundary between telomere and subtelomere. In order to differentiate between the various type of telomere read we must first understand how reads differ from the telomere sequence and whether these differences are genuine biological perturbations or the result of sequencing error.

Lastly, we use the ratio of complete to boundary read-pairs in conjunction with insert length distribution to estimate the underlying telomere length that produced the observed complete to boundary ratio.

### Defining error in telomere reads

Key to the process of identifying sequencing error is identifying loci within reads that do not match the expected telomere sequence. We shall refer to these as “mismatching loci”. Telomeres are extremely repetitive stretches of DNA. This repetition of sequence allows us to imagine a hypothetical telomere sequence and then to compare reads to the hypothetical sequence to find differences. In order to account for insertions and deletions in the sequencing reads (both biological and as a result of sequencing error) we use a method of fragmentary local alignment. Reads that suffer few mismatches, and those mismatches at loci with low Phred scores, likely represent complete telomere sequences.

**Figure 7:**
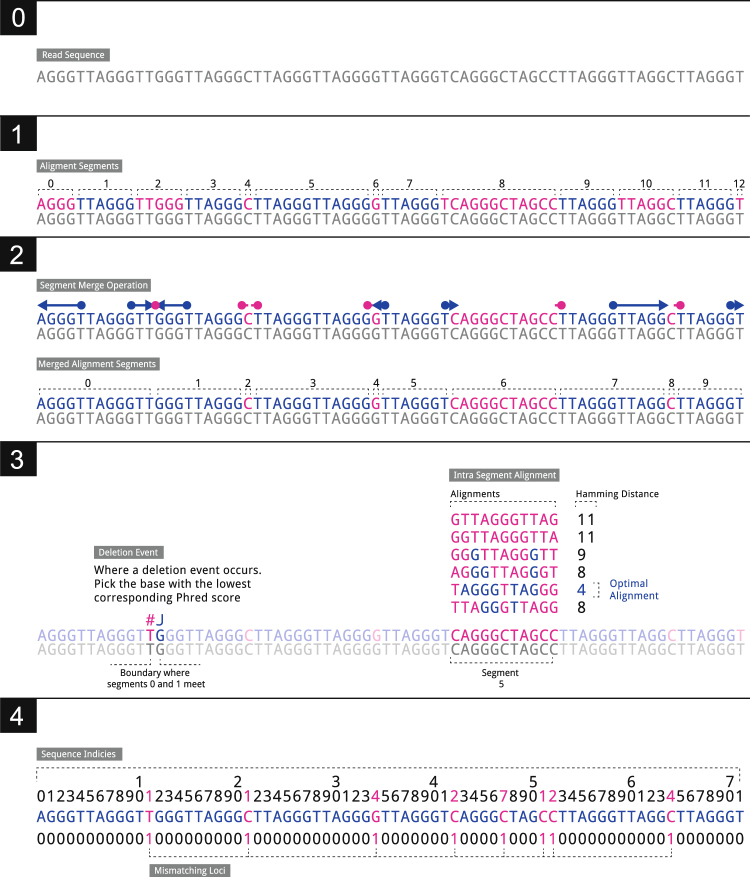
The algorithm that determines the indices of divergence from the telomere sequence. **0**: We observe a sequencing read **1**: We split the read into ‘segments’ (11 in total in our example) such that each segment is a substring of the original sequence and that every other segment consists of unbroken telomere sequence. In our example we see that segments 1,3,5,7,9,11 contain unbroken telomere sequence. **2**: Each segment containing a telomere hexamer is ‘expanded‘ to capture the full extent of the surrounding telomere sequence. The number of segments is reduced by 2. **3**: When two segments both containing the telomere hexamer are adjacent after Step 2 this indicates a deletion event. We take the loci with the lowest corresponding Phred score. For any segment that does not contain a telomere hexamer and where the length of the segment is greater or equal to 4 apply we conduct a basic alignment of all possible telomere offset telomere sequences. The telomere sequence with the lowest Hamming distance is taken as a local alignment for that segment. Where two alignments are equal the one with the lowest average Phred score is preferred. **4**: Sequence loci that are not in a complete hexamer or were mismatched in the Hamming alignment step are taken as mismatching loci. **m** for this example is given in the final line of the diagram.

**Figure 8:**
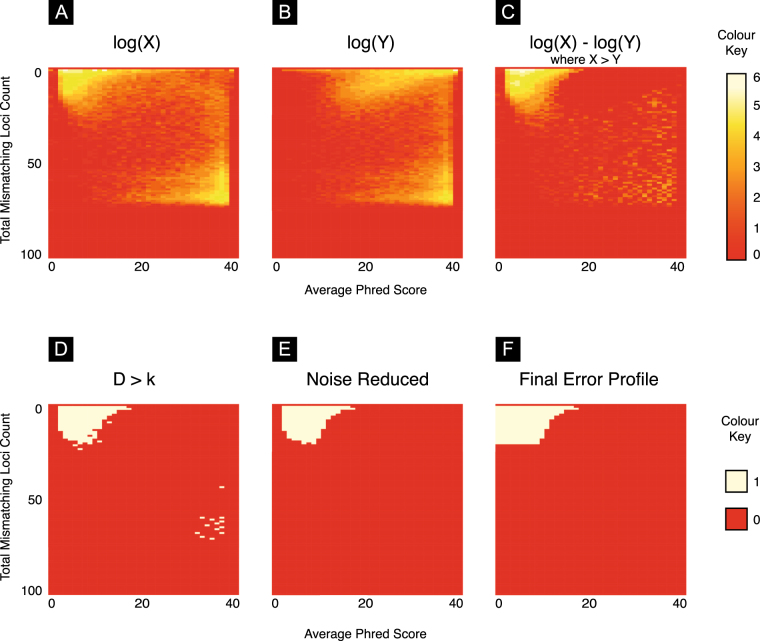
(**A**) A heatmap of the joint distribution of Phred scores a mismatching loci and the number of mismatching loci (**X**). The intensities in the top left corner of the heatmap indicate an association between fewer mismatches and lower phred scores. We observe that the maximum mismatching loci is commonly ~75% of the read length. This effect is caused by non-telomere reads match a the telomere sequence simply by chance (**B**) A heatmap of the joint distribution of random loci in reads and the associated phred score (**Y**). We note that the joint distirubtion of reads in the upper half of the matrix is different to that in **X** while the lower portion is identical. (**C**) The difference between **X** and **Y**. Referred to as **D** in the text. (**D**) A binary heatmap showing all cells in **D** that are greater than the threshold *k*. We note the preponderance of cells in the upper left hand corner of the figure (**E**) We remove noise from the figure using the methods detailed in (Supplementary Algorithm 1) (**F**) We apply a final rule to ensure cells associated with low Phred scores are captured in the error profile (Supplementary Algorithm 2).

Since mismatch loci that represent sequencing errors should be associated with lower Phred scores, we first observe the empirical joint distribution of Phred scores at mismatching loci (as determined by the algorithm shown in Fig. 7), and number of mismatching loci across the BAM file (Fig. 8A) before constructing the equivalent distribution for loci chosen at random within the reads (Fig. 8B). We find that reads with few mismatches and low Phred scores (complete telomere sequences suffering from sequencing error) are over-represented in the empirical data set.

We define *P*_*max*_ and *P*_*min*_ as the global maximum and minimum observed Phred score across all reads, and (*L*) as the read length used.

We let *N* represent the total number of reads in the TELBAM such that $\mathrm{\{0},1,n,\mathrm{...},N-\mathrm{1\}}$ are indices representing each read. Values associated with the *n*^*th*^ read are denoted with a superscript (*n*). For example, the vector of Phred scores associated with the L locations in read n is denoted ${{\bf{p}}}^{(n)}=\{{p}_{0}^{(n)},{p}_{1}^{(n)},\mathrm{...},{p}_{L-1}^{(n)}\}$. For the *n*^*th*^ read, let ${m}^{(n)}$ be a random vector in the space ${\mathrm{\{0},\mathrm{1\}}}^{L}$ such that a 1 is found at each loci in the read that does not agree with the telomere sequence. In the case that the sequence is comprised of perfect telomere sequence then the vector should sum to zero. The method for obtaining ${m}^{(n)}$ via an fragmentary alignment method is shown in Fig. 7.

Then define *z*^*n*^ (the number of mismatches for read n), and *λ*^*n*^ (the average Phred score at mismatches in read n) as:

1 $$\begin{array}{rcl}{z}^{n} & = & \sum _{i=0}^{L-1}{{\bf{m}}}_{i}^{(n)}\\ {\lambda }^{n} & = & \lfloor \frac{\sum _{i=0}^{L-1}{{\bf{m}}}_{i}^{(n)}{{\bf{p}}}_{i}^{(n)}}{{z}^{(n)}}\rfloor -{P}_{min}\end{array}$$

We then define an indicator function

2 $$\begin{array}{ll}\mathrm{1(}\lambda ,z,i,j) & :=\{\begin{array}{cc}1 & {\rm{if}}\,\lambda =i\wedge z=j,\\ 0 & {\rm{if}}\,\lambda \ne i\vee z\ne j\mathrm{.}\end{array}\end{array}$$

So that a matrix **X** takes the form,

3 $$\begin{array}{l}{x}_{ij}=\sum _{n\mathrm{=0}}^{N-1}\mathrm{1(}{\lambda }^{(n)},{z}^{(n)},i,j)\end{array}$$

Where $i\in \mathrm{\{0},\mathrm{...},{P}_{max}-{P}_{min}\}$ and $j\in \mathrm{\{0},\mathrm{...},L-\mathrm{1\}}$. Thus each *x*_*ij*_ in **X** records the number of reads with the relevant $\lambda$ and $z$ contained within the TELBAM and is depicted in Fig. 8A.

Where **X** captures information about the average Phred score (${\lambda }^{(n)}$) at ${z}^{(n)}$ mismatching loci, we seek to create an equivalent matrix **Y** about the average Phred score at ${z}^{(n)}$ *random* loci in the ${n}^{th}$ read.

For the ${n}^{th}$ read, let ${r}^{(n)}$ be a random vector in the space ${\mathrm{\{0,1\}}}^{L}$ such that ${\sum }_{k\mathrm{=1}}^{L}{r}_{k}^{(n)}={z}^{(n)}$. That is, a vector for which the non-zero entries identify ${z}^{(n)}$ random loci within the read.

So that,

4 $$\begin{array}{rcl}{\mu }^{(n)} & = & \lfloor \frac{\sum _{i\mathrm{=1}}^{L}{{\bf{r}}}_{i}^{(n)}{{\bf{p}}}_{i}}{{z}^{(n)}}\rfloor -{P}_{min}\end{array}$$

Thus,

5 $$\begin{array}{rcl}\mathrm{1(}\mu ,z,i,j)\,\,\,\,: & = & \{\begin{array}{cc}1 & {\rm{if}}\,\mu =i\wedge z=j,\\ 0 & {\rm{if}}\,\mu \ne i\vee z\ne j\mathrm{.}\end{array}\\ {y}_{ij} & = & \sum _{n\mathrm{=0}}^{N-1}\mathrm{1(}{\mu }^{(n)},{z}^{(n)},i,j)\end{array}$$

As before, $i\in \mathrm{\{0},\mathrm{...},{P}_{max}-{P}_{min}\}$ and j $\in \mathrm{\{0},\mathrm{...},L-\mathrm{1\}}$.

When we plot the matrices **X** (Fig. 8A) and **Y** (Fig. 8B) as heat maps we typically see that there is a striking difference in their composition. The heatmap for **X** shows an intensity in the upper left hand corner pertaining to reads with low Phred scores at mismatching loci. This hotspot is missing from the **Y** heatmap. We interpret this region as representing telomere reads affected by sequencing error that we wish to capture in our length estimation process.

We find the difference between the two matrices:

6 $$\begin{array}{l}{\bf{D}}={\bf{X}}-{\bf{Y}}\end{array}$$

We plot values of **D** > 0 as a heatmap in 8C. To capture cells that contain more reads than we would expect at random we define a mask **E**. **E** is defined such that:

7 $$\begin{array}{l}{e}_{ij}=(\begin{array}{cc}1 & {\rm{if}}\,{d}_{ij} > k,\\ 0 & {\rm{if}}\,{d}_{ij}\le k\mathrm{.}\end{array}\end{array}$$

Where *k* is $max\{{{\bf{D}}}_{ij}\}$ for all values where $\frac{1}{2}p < i\le p$ and $\frac{1}{2}L < j\le L$. This matrix is depicted as a heatmap in Fig. 8D.

We note that the mask depicted in Fig. 8D has gaps that appear as a result of using *k* as a threshold. We apply the procedure detailed in Supplementary Algorithm 1 in order to remove noise from the error profile. The results of applying this procedure are shown in Fig. 8E. We conclude by applying the operation described in Supplementary Algorithm 2 and shown in Fig. 8F. This is the final matrix and is provided to the read classification procedure shown in Supplementary Algorithm 3 as **E**. All reads falling within the area by the error profile are counted as fully telomeric suffering from sequencing error.

Our definitive definition of a fully telomeric read is a read where 90% of the the sequence is telomere or the read falls into the error profile (See Supplementary Algorithm 3). In practice we observe that using a threshold above 90% leads to decreased accuracy. It is possible that this is indicative of genuine telomere heterogeneity but further study is required to understand this phenomenon.

### Categorising telomere read types

**Figure 9:**
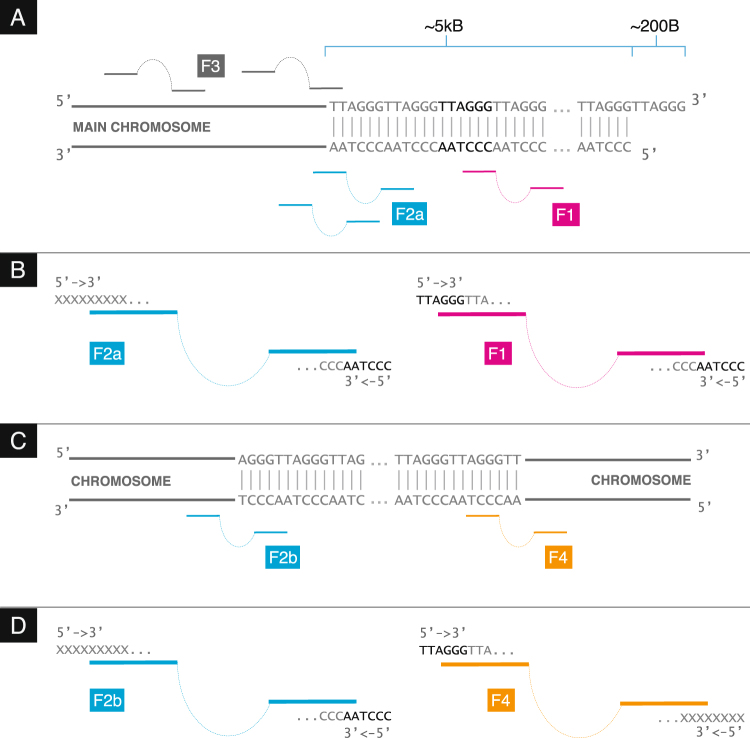
(**A**) The read-pair types at the boundary between telomere and subtelomere. F2a reads stem from the boundary whereas F1 reads stem from anywhere within the telomere proper. F3 are reads where neither read in the pair is complete telomere (**B**) Detail of the F1 and F2a read types. F1 read-pairs are comprised of two complete telomere reads. F2a read-pairs are comprised of a read-pair where one read is complete telomere and the other is not. Crucially, the complete telomere read is comprised of CCCTAA (**C**) The read-pair types at an ITR. (**D**) Detail of the F2b and F4 read types. Note that the F2b is physical indistinguishable from an F2a read. An F4 read is read-pair where one read is complete telomere and the other is not. The complete end is comprised of TTAGGG.

Once we have adequately described sequencing error we now classify each read-pair. In this section we describe the step that allows us to sort read-pairs into ‘complete’ read-pairs (denoted F1 reads in Fig. 9 - both reads of the pair lying wholly within the telomere) and boundary (F2a - exactly one read of the pair lying wholly within the telomere) reads.

The Telomerecat length estimation method requires that all read pairs are sorted into four categories: F1, F2, F3 F4. Examples of each read type are given in Fig. 9. Pseudocode for categorisation of reads is given in Supplementary Algorithm 3.

The read categorisation process is crucial to Telomerecat’s ability to filter interstitial reads. As we see in Fig. 9, the F2 category contains read pairs where one end consists of the canonical CCCTAA telomeric repeat and the other does not. Read pairs that meet this criteria can be found both at the boundary between the telomere and the rest of the genome, and on one side of an ITR. We refer to these two distinct cases as F2a and F2b, but we cannot directly observe the number of F2a or F2b read pairs; the orientation and sequence content of the read types are identical. However, the directional nature of WGS allows us to identify read pairs spanning the other boundary between an ITR and the genome. For such read pairs the telomere-like end will be read as TTAGGG, allowing us to easily distinguish them. We categorise these as F4 read pairs in Fig. 9. Read pairs in this category should only be found at ITR boundaries, as the chromosome does not continue beyond truly telomeric read pairs. We can use this fact, combined with the observation that on average, within a sequencing experiment, there should be a corresponding F2b for each F4, to deduce the amount of F2a reads. So it follows that.

8 $$\begin{array}{rcl}F2b & \equiv  & F4\\ F2a & = & F2-F2b\end{array}$$

F4 reads give us an estimate of ITR reads, so subtracting F4 from F2 we are left with a count of reads F2 for which there was no corresponding F4. We posit that this is the count of reads on the boundary between telomere and subtelomere.

This method allows us to attain an estimate of F2a without filtering reads based on any upstream processing or any sequence structure beyond a distinction between “complete” and “incomplete” (see Supplementary Algorithm 3).

### Using cohort wide information to correct error in F2a counts

We observe that in some cases it is useful to normalise a cohort’s *F*2*a* count based on information from other samples in the batch. What follows is a method for adjusting F2a using a weighted average.

Let *C* be the total number of TELBAMs in a batch provided to Telomerecat. Such that subscript *c* represents a value relevant to any individual TELBAM. Let $\theta =\frac{F2a}{F2+F4}$ such that *θ*^*exp*^ is the average *θ* observed across all TELBAMs in a cohort and ${\theta }_{c}^{obs}$ is the observed value of *θ* with in a particular TELBAM.

9 $$\begin{array}{rcl}{\theta }^{exp} & = & \frac{\sum _{c=1}^{C}{\theta }_{c}^{obs}}{C}\\ {\theta }_{c}^{cor} & = & \frac{{\theta }_{c}^{obs}\cdot {\psi }_{c}+{\theta }^{exp}\cdot w}{{\psi }_{c}\cdot w}\end{array}$$

Where *w* is a predetermined weight of 3. *ψ* for any given TELBAM is obtained as follows.

10 $$\begin{array}{rcl}{\mu }_{c} & = & \frac{\sum _{i=1}^{\frac{2}{5}p}\sum _{j=1}^{L}Xij}{L\cdot (\frac{2}{5}p)}\\ {\sigma }_{c} & = & \frac{\sum _{i=1}^{\frac{2}{5}p}\sum _{j=1}^{L}{(Xij-{\mu }_{c})}^{2}}{L\cdot (\frac{2}{5}p)}\\ {\psi }_{c} & = & \frac{{\sigma }_{c}}{{\mu }_{c}}\end{array}$$

So it follows that the adjusted value of F2a is given as ${\theta }^{cor}\cdot (F2+F\mathrm{4)}$.

### Estimating length from read pair categories

The final step of the telomere length estimation process involves converting a ratio of *F*1:*F*2*a* read counts into an estimation of length. We achieve this by simulating telomere length under the observation of counts for F1, F2a and the fragment size. Psuedocode for the simulation is given in Algorithm 1.

**Algorithm 1:**
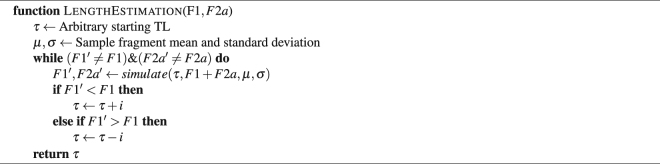
Telomerecat length estimation simulation algorithm.

### Batch effect correction when multiple sequencing platforms are used

Our observation has been that estimates from the HiSeqX platform are shorter on average than estimates from the HiSeq. 2000 platform. We have also observed that samples sequenced on the HiSeqX platform show lower scores in quality assessment. To account for this effect we propose that a mean correction should be applied to estimates from the HiSeqX platform.

### Data Availability

1. The Twins UK10K sequencing data are available from the EGA repository (accession ID: EGAD00001000194) but restrictions apply to the availability of these data, which were used under license for the current study, and so are not publicly available. Data are however available upon reasonable request to datasharing@sanger.ac.uk and with permission of Twins UK10K.
2. The MSC sequencing datasets analysed during the current study are available in the NCBI SRA repository under accession ID SRP032359, https://www.ncbi.nlm.nih.gov/sra/?term=SRP032359.
3. The HCC sequencing sequencing data are available from the EGA repository (accession ID: EGAD00001001995) but restrictions apply to the availability of these data, which were used under license for the current study, and so are not publicly available. Data are however available upon reasonable request to qiuzhixin@sibcb.ac.cn.
4. The repeated measurement sequencing data are available from the EGA repository (accession ID: EGAD00001003809) but restrictions apply to the availability of these data, which were used under license for the current study, and so are not publicly available. Data are however available upon reasonable request to Kathleen Stirrups (nihr_dac@medschl.cam.ac.uk) and with permission of NIHR BioResource - Rare Diseases.
5. The mice sequencing datasets analysed during the current study are available from the mouse genome project website repository, http://www.sanger.ac.uk/science/data/mouse-genomes-project.

## Electronic supplementary material

telomerecat-supplementary-information-file

## References

[1] O’Sullivan RJ, Karlseder J (2010). Telomeres: protecting chromosomes against genome instability. Nat. Rev. Mol. Cell Biol..

[2] Blackburn EH, Epel ES, Lin J (2015). Human telomere biology: A contributory and interactive factor in aging, disease risks, and protection. Science.

[3] Maciejowski J, de Lange T (2017). Telomeres in cancer: tumour suppression and genome instability. Nat. Rev. Mol. Cell Biol..

[4] Blasco MA (2005). Telomeres and human disease: ageing, cancer and beyond. Nat. Rev. Genet..

[5] MacArthur J (2017). The new NHGRI-EBI Catalog of published genome-wide association studies (GWAS Catalog). Nucleic Acids Res..

[6] Barthel FP (2017). Systematic analysis of telomere length and somatic alterations in 31 cancer types. Nat. Genet..

[7] Hanahan D, Weinberg RA (2011). Hallmarks of cancer: the next generation. Cell.

[8] Castle JC (2010). DNA copy number, including telomeres and mitochondria, assayed using next-generation sequencing. BMC Genomics.

[9] Parker M (2012). Assessing telomeric DNA content in pediatric cancers using whole-genome sequencing data. Genome Biol..

[10] Ding Z (2014). Estimating telomere length from whole genome sequence data. Nucleic Acids Res..

[11] Robles-Espinoza CD (2014). POT1 loss-of-function variants predispose to familial melanoma. Nat. Genet..

[12] Zheng S (2016). Comprehensive Pan-Genomic Characterization of Adrenocortical Carcinoma. Cancer Cell.

[13] Zack TI (2013). Pan-cancer patterns of somatic copy number alteration. Nat. Genet..

[14] Feuerbach, L. *et al*. Telomerehunter: telomere content estimation and characterization from whole genome sequencing data. *bioRxiv*, http://biorxiv.org/content/early/2016/07/23/065532 (2016).

[15] Nersisyan L, Arakelyan A (2015). Computel: computation of mean telomere length from whole-genome next-generation sequencing data. PLoS one.

[16] Lee M (2017). Comparative analysis of whole genome sequencing-based telomere length measurement techniques. Methods.

[17] Bolzan AD, Bianchi MS (2006). Telomeres, interstitial telomeric repeat sequences, and chromosomal aberrations. Mutat. Res..

[18] Riethman H (2004). Mapping and initial analysis of human subtelomeric sequence assemblies. Genome Res..

[19] Gutierrez-Rodrigues F, Santana-Lemos BA, Scheucher PS, Alves-Paiva RM, Calado RT (2014). Direct comparison of flow-FISH and qPCR as diagnostic tests for telomere length measurement in humans. PLoS ONE.

[20] Valdes AM (2005). Obesity, cigarette smoking, and telomere length in women. Lancet.

[21] Moayyeri A, Hammond CJ, Hart DJ, Spector TD (2013). The UK Adult Twin Registry (TwinsUK Resource). Twin Res Hum Genet.

[22] Cai J (2014). Whole-genome sequencing identifies genetic variances in culture-expanded human mesenchymal stem cells. Stem Cell Reports.

[23] Minguell JJ, Erices A, Conget P (2001). Mesenchymal stem cells. Exp. Biol. Med. (Maywood).

[24] Zimmermann S (2003). Lack of telomerase activity in human mesenchymal stem cells. Leukemia.

[25] Graakjaer J, Christensen R, Kolvraa S, Serakinci N (2007). Mesenchymal stem cells with high telomerase expression do not actively restore their chromosome arm specific telomere length pattern after exposure to ionizing radiation. BMC Molecular Biology.

[26] Samsonraj RM (2013). Telomere length analysis of human mesenchymal stem cells by quantitative PCR. Gene.

[27] Marion RM (2009). Telomeres acquire embryonic stem cell characteristics in induced pluripotent stem cells. Cell Stem Cell.

[28] Qiu Z (2016). Hepatocellular carcinoma cell lines retain the genomic and transcriptomic landscapes of primary human cancers. Sci Rep.

[29] Sung WK (2012). Genome-wide survey of recurrent HBV integration in hepatocellular carcinoma. Nat. Genet..

[30] Nault JC, Zucman-Rossi J (2016). TERT promoter mutations in primary liver tumors. Clin Res Hepatol Gastroenterol.

[31] Yujing Z, Jing S, Ming-Whei, Yu Po-Huang L, Regina MS (2007). Telomere length in hepatocellular carcinoma and paired adjacent non-tumor tissues by quantitative pcr. Cancer Investigation.

[32] Aubert G, Hills M, Lansdorp PM (2012). Telomere length measurement-caveats and a critical assessment of the available technologies and tools. Mutat. Res..

[33] Kipling D, Cooke HJ (1990). Hypervariable ultra-long telomeres in mice. Nature.

[34] Keane TM (2011). Mouse genomic variation and its effect on phenotypes and gene regulation. Nature.

[35] Callicott RJ, Womack JE (2006). Real-time PCR assay for measurement of mouse telomeres. Comp. Med..

[36] Hemann MT, Greider CW (2000). Wild-derived inbred mouse strains have short telomeres. Nucleic Acids Res..

[37] Zhu L (1998). Telomere length regulation in mice is linked to a novel chromosome locus. Proc. Natl. Acad. Sci. USA.

[38] Farmery, J. H. P: Parallel processing for BAM files (2017). www.github.com/user/jhrf. [Online; accessed 21-April-2017].

