# Publisher Correction: Telomerecat: A ploidy-agnostic method for estimating telomere length from whole genome sequencing data

**DOI:** 10.1038/s41598-018-31524-0

**Published:** 2018-09-03

**Authors:** James H. R. Farmery, Mike L. Smith, NIHR BioResource - Rare Diseases, Aarnoud Huissoon, Abigail Furnell, Adam Mead, Adam P. Levine, Adnan Manzur, Adrian Thrasher, Alan Greenhalgh, Alasdair Parker, Alba Sanchis-Juan, Alex Richter, Alice Gardham, Allan Lawrie, Aman Sohal, Amanda Creaser-Myers, Amy Frary, Andreas Greinacher, Andreas Themistocleous, Andrew J. Peacock, Andrew Marshall, Andrew Mumford, Andrew Rice, Andrew Webster, Angie Brady, Ania Koziell, Ania Manson, Anita Chandra, Anke Hensiek, Anna Huis in’t Veld, Anna Maw, Anne M. Kelly, Anthony Moore, Anton Vonk Noordegraaf, Antony Attwood, Archana Herwadkar, Ardi Ghofrani, Arjan C. Houweling, Barbara Girerd, Bruce Furie, Carmen M. Treacy, Carolyn M. Millar, Carrock Sewell, Catherine Roughley, Catherine Titterton, Catherine Williamson, Charaka Hadinnapola, Charu Deshpande, Cheng-Hock Toh, Chiara Bacchelli, Chris Patch, Chris Van Geet, Christian Babbs, Christine Bryson, Christopher J. Penkett, Christopher J. Rhodes, Christopher Watt, Claire Bethune, Claire Booth, Claire Lentaigne, Coleen McJannet, Colin Church, Courtney French, Crina Samarghitean, Csaba Halmagyi, Daniel Gale, Daniel Greene, Daniel Hart, David Allsup, David Bennett, David Edgar, David G. Kiely, David Gosal, David J. Perry, David Keeling, David Montani, Debbie Shipley, Deborah Whitehorn, Debra Fletcher, Deepa Krishnakumar, Detelina Grozeva, Dinakantha Kumararatne, Dorothy Thompson, Dragana Josifova, Eamonn Maher, Edwin K. S. Wong, Elaine Murphy, Eleanor Dewhurst, Eleni Louka, Elisabeth Rosser, Elizabeth Chalmers, Elizabeth Colby, Elizabeth Drewe, Elizabeth McDermott, Ellen Thomas, Emily Staples, Emma Clement, Emma Matthews, Emma Wakeling, Eric Oksenhendler, Ernest Turro, Evan Reid, Evangeline Wassmer, F. Lucy Raymond, Fengyuan Hu, Fiona Kennedy, Florent Soubrier, Frances Flinter, Gabor Kovacs, Gary Polwarth, Gautum Ambegaonkar, Gavin Arno, Gavin Hudson, Geoff Woods, Gerry Coghlan, Grant Hayman, Gururaj Arumugakani, Gwen Schotte, H. Terry Cook, Hana Alachkar, Hana Lango Allen, Hana Lango-Allen, Hannah Stark, Hans Stauss, Harald Schulze, Harm J. Boggard, Helen Baxendale, Helen Dolling, Helen Firth, Henning Gall, Henry Watson, Hilary Longhurst, Hugh S. Markus, Hugh Watkins, Ilenia Simeoni, Ingrid Emmerson, Irene Roberts, Isabella Quinti, Ivy Wanjiku, J. Simon R. Gibbs, James Thaventhiran, James Whitworth, Jane Hurst, Janine Collins, Jay Suntharalingam, Jeanette Payne, Jecko Thachil, Jennifer M. Martin, Jennifer Martin, Jenny Carmichael, Jesmeen Maimaris, Joan Paterson, Joanna Pepke-Zaba, Johan W. M. Heemskerk, Johanna Gebhart, John Davis, John Pasi, John R. Bradley, John Wharton, Jonathan Stephens, Julia Rankin, Julie Anderson, Julie Vogt, Julie von Ziegenweldt, Karola Rehnstrom, Karyn Megy, Kate Talks, Kathelijne Peerlinck, Katherine Yates, Kathleen Freson, Kathleen Stirrups, Keith Gomez, Kenneth G. C. Smith, Keren Carss, Kevin Rue-Albrecht, Kimberley Gilmour, Larahmie Masati, Laura Scelsi, Laura Southgate, Lavanya Ranganathan, Lionel Ginsberg, Lisa Devlin, Lisa Willcocks, Liz Ormondroyd, Lorena Lorenzo, Lorraine Harper, Louise Allen, Louise Daugherty, Manali Chitre, Manju Kurian, Marc Humbert, Marc Tischkowitz, Maria Bitner-Glindzicz, Marie Erwood, Marie Scully, Marijke Veltman, Mark Caulfield, Mark Layton, Mark McCarthy, Mark Ponsford, Mark Toshner, Marta Bleda, Martin Wilkins, Mary Mathias, Mary Reilly, Maryam Afzal, Matthew Brown, Matthew Rondina, Matthew Stubbs, Matthias Haimel, Melissa Lees, Michael A. Laffan, Michael Browning, Michael Gattens, Michael Richards, Michel Michaelides, Michele P. Lambert, Mike Makris, Minka De Vries, Mohamed Mahdi-Rogers, Moin Saleem, Moira Thomas, Muriel Holder, Mélanie Eyries, Naomi Clements-Brod, Natalie Canham, Natalie Dormand, Natalie Van Zuydam, Nathalie Kingston, Neeti Ghali, Nichola Cooper, Nicholas W. Morrell, Nigel Yeatman, Noémi Roy, Olga Shamardina, Omid S. Alavijeh, Paolo Gresele, Paquita Nurden, Patrick Chinnery, Patrick Deegan, Patrick Yong, Patrick Yu-Wai-Man, Paul A. Corris, Paul Calleja, Paul Gissen, Paula Bolton-Maggs, Paula Rayner-Matthews, Pavandeep K. Ghataorhe, Pavel Gordins, Penelope Stein, Peter Collins, Peter Dixon, Peter Kelleher, Phil Ancliff, Ping Yu, R. Campbell Tait, Rachel Linger, Rainer Doffinger, Rajiv Machado, Rashid Kazmi, Ravishankar Sargur, Remi Favier, Rhea Tan, Ri Liesner, Richard Antrobus, Richard Sandford, Richard Scott, Richard Trembath, Rita Horvath, Rob Hadden, Rob V. MackenzieRoss, Robert Henderson, Robert MacLaren, Roger James, Rohit Ghurye, Rosa DaCosta, Rosie Hague, Rutendo Mapeta, Ruth Armstrong, Sadia Noorani, Sai Murng, Saikat Santra, Salih Tuna, Sally Johnson, Sam Chong, Sara Lear, Sara Walker, Sarah Goddard, Sarah Mangles, Sarah Westbury, Sarju Mehta, Scott Hackett, Sergey Nejentsev, Shahin Moledina, Shahnaz Bibi, Sharon Meehan, Shokri Othman, Shoshana Revel-Vilk, Simon Holden, Simon McGowan, Simon Staines, Sinisa Savic, Siobhan Burns, Sofia Grigoriadou, Sofia Papadia, Sofie Ashford, Sol Schulman, Sonia Ali, Soo-Mi Park, Sophie Davies, Sophie Stock, Souad Ali, Sri V. V. Deevi, Stefan Gräf, Stefano Ghio, Stephen J. Wort, Stephen Jolles, Steve Austin, Steve Welch, Stuart Meacham, Stuart Rankin, Suellen Walker, Suranjith Seneviratne, Susan Holder, Suthesh Sivapalaratnam, Sylvia Richardson, Taco Kuijpers, Taco W. Kuijpers, Tadbir K. Bariana, Tamam Bakchoul, Tamara Everington, Tara Renton, Tim Young, Timothy Aitman, Timothy Q. Warner, Tom Vale, Tracey Hammerton, Val Pollock, Vera Matser, Victoria Cookson, Virginia Clowes, Waseem Qasim, Wei Wei, Wendy N. Erber, Willem H. Ouwehand, William Astle, William Egner, Wojciech Turek, Yvonne Henskens, Yvonne Tan, Andy G. Lynch

**Affiliations:** 1 Cancer Research UK Cambridge Institute, University of Cambridge, Li Ka Shing Centre, Robinson Way, Cambridge, CB2 0RE UK; 2 European Molecular Biology Laboratory (EMBL), Genome Biology Unit, 69117 Heidelberg, Germany; 3 School of Mathematics and Statistics/School of Medicine, University of St Andrews, St Andrews, Fife, KY16 9SS UK; 4 Birmingham Heartlands, Bordesley Green E, Birmingham, B9 5SS UK; 5 University of Cambridge, The Old Schools, Trinity Lane, Cambridge, CB2 1TN UK; 6 Oxford University Hospitals NHS Foundation Trust, John Radcliffe Hospital, Headley Way, Headington, Oxford, OX3 9DU UK; 7 University of Oxford, University Offices, Wellington Square, Oxford, OX1 2JD UK; 8 Centre for Nephrology, University College London, UCL Medical School, Rowland Hill Street, London, NW3 2PF UK; 9 Great Ormond Street Hospital for Children NHS Foundation Trust, Great Ormond Street, London, WC1N 3JH UK; 10 UCL Great Ormond Street Institute of Child Health, 30 Guilford St, London, WC1N 1EH UK; 11 Newcastle Freeman Hospital, Freeman Rd, High Heaton Newcastle upon Tyne, NE7 7DN UK; 12 Cambridge University Hospitals NHS Foundation Trust, Addenbrookes Hospital, Hills Rd, Cambridge, CB2 0QQ UK; 13 University Hospitals Birmingham, Mindelsohn Way, Edgbaston Birmingham, B15 2TH UK; 14 Sheffield CRF, Royal Hallamshire, Royal Hallamshire Hospital, Glossop Road, Sheffield, S10 2JF UK; 15 Birmingham Children’s Hospital NHS Foundation Trust, Steelhouse Ln, Birmingham, B4 6NH UK; 16 Institute for Immunology and Transfusion Medicine, Ernst-Moritz-Arndt-University of Greifswald, Domstraße 11, 17489 Greifswald, Germany; 17 Golden Jubilee National Hospital, Agamemnon St, Clydebank, G81 4DY UK; 18 Salford Royal NHS Foundation Trust, Stott Ln, Salford, M6 8HD UK; 19 University Hospitals Bristol NHS Foundation Trust, Trust Headquarters, Marlborough Street, Bristol, BS1 3NU UK; 20 University of Bristol, Senate House, Tyndall Avenue, Bristol, BS8 1TH UK; 21 Imperial College, Kensington London, SW7 2AZ UK; 22 Moorfields Eye Hospital NHS Foundation Trust, 162 City Road, London, EC1V 2PD UK; 23 University College London, Gower St, Bloomsbury, London, WC1E 6BT UK; 24 London North West Healthcare NHS Trust, Northwick Park Hospital, Watford Road, Harrow, HA1 3UJ UK; 25 Guy’s and St Thomas’ NHS Foundation Trust, St Thomas’ Hospital, Westminster Bridge Road, London, SE1 7EH UK; 26 VU University Medical Center, De Boelelaan, 1117, 1081 HV Amsterdam, Netherlands; 27 University of Giessen, Ludwigstraße 23, 35390 Gießen, Germany; 28 University of South Paris, 15 Rue Georges Clemenceau, 91400 Orsay, France; 29 Beth Israel Deaconess Medical Centre, Harvard Medical School, 330 Brookline Ave, Boston, MA 02215 USA; 30 Department of Medicine, University of Cambridge, Addenbrooke’s Hospital, Hills Rd, Cambridge, CB2 0SP UK; 31 Imperial College Healthcare NHS Trust, The Bays, St Mary’s Hospital, South Wharf Road, London, W2 1NY UK; 32 Scunthorpe General Hospital, Cliff Gardens, Scunthorpe, DN15 7BH UK; 33 Haemophilia Centre, Kent & Canterbury Hospital, East Kent Hospitals University Foundation Trust, Ethelbert Road, Canterbury, Kent TN24 OLZ UK; 34 King’s College, Strand, London, WC2R 2LS UK; 35 The Roald Dahl Haemophilia Centre, Royal Liverpool Hospital, Prescot St, Liverpool, L7 8XP UK; 36 Department of Cardiovascular Sciences, Center for Molecular and Vascular Biology, University of Leuven, Oude Markt 13, 3000 Leuven, Belgium; 37 Imperial and Hammersmith Hospitals, Du Cane Rd, Shepherd’s Bush, London, W12 0HS UK; 38 Plymouth Hopsital, Derriford Road, Crownhill, Clymouth, Devon PL6 8DH UK; 39 Department of Haematology, University of Cambridge, Wellcome Trust Mrc Bldg, Addenbrookes Hospital, Hills Rd, Cambridge, CB2 0XY UK; 40 MRC-BSU, Cambridge Institute of Public Health, Forvie Site, Robinson Way, Cambridge Biomedical Campus, Cambridge, CB2 0SR UK; 41 The Royal London Hospital, Barts Health NHS Trust, Whitechapel Rd, Whitechapel, E1 1BB UK; 42 Department of Haematology, Castle Hill Hospital, Hull and East Yorkshire NHS Foundation Trust, Castle Road, Cottingham, HU16 5JQ UK; 43 Royal Hospitals Belfast, Trust Headquarters, A Floor, Belfast City Hospital, Lisburn Road, Belfast, BT9 7AB UK; 44 Oxford Haemophilia and Thrombosis Centre, Oxford University Hospitals NHS Trust, The Churchill Hospital, Churchill Hospital, Oxford, OX3 7LE UK; 45 University of Cambridge (CIMR Medical Genetics), Cambridge Institute for Medical Research, University of Cambridge, Cambridge Biomedical Campus, Wellcome Trust/MRC Building, Hills Road, Cambridge, CB2 0XY UK; 46 National Renal Complement Therapeutics Centre, Newcastle University, Royal Victoria Infirmary - Victoria Wing, Newcastle upon Tyne, NE1 4LP UK; 47 University College London Hospitals NHS Foundation Trust, 235 Euston Rd, Bloomsbury London, NW1 2BU UK; 48 Royal Hospital for Children, NHS Greater Glasgow and Clyde, 1345 Govan Rd, Glasgow, G51 4TF UK; 49 Nottingham University Hospitals NHS Trust, Hucknall Rd, Nottingham, NG5 1PB UK; 50 The National Hospitals for Neurology and Neurosurgery, UCLH and UCL, National Hospital for Neurology & Neurosurgery, Queen Square London, WC1N 3BG UK; 51 Hopital St Louis, 1 Avenue Claude Vellefaux, 75010 Paris, France; 52 University of Sorbonne, 75005 Paris, France; 53 University of Graz, 8010, Universit ätspl. 3, 8010 Graz, Austria; 54 Papworth Hospital, Papworth Everard, Cambridge, CB23 3RE UK; 55 Royal Free Hospital, Pond St, Hampstead London, NW3 2cvG UK; 56 Epsom & St Helier University Hospitals NHS Trust, Wrythe Ln, Sutton Carshalton, SM5 1AA UK; 57 Leeds Teaching Hospitals NHS Foundation Trust, Great George Street, Leeds, West Yorkshire LS1 3EX UK; 58 Centre for Complement and Inflammation Research, Imperial College, London, SW7 2AZ UK; 59 Lehrstuhl für Experimentelle Biomedizin, Universitätsklinikum Würzburg, Josef-Schneider-Straße 2, 97080 Würzburg, Germany; 60 Aberdeen Royal Infirmary, Foresterhill, Aberdeen, AB25 2ZN UK; 61 Barts Health NHS Trust, Turner St, Whitechapel London, E1 1BB UK; 62 Newcastle University, Newcastle upon Tyne, NE1 7RU UK; 63 Sapienza Universita di Roma, Piazzale Aldo Moro, 5, 00185 Roma, RM Italy; 64 National Heart & Lung Institute, Imperial College, Dovehouse Street, London, SW3 6LR UK; 65 Royal United Bath Hospitals, Combe Park, Avon, BA1 3NG UK; 66 Department of Haematology, Sheffield Children’s Hospital NHS Foundation Trust, Western Bank Sheffield, S10 2TH UK; 67 Haematology Department, Manchester Royal Infirmary, Oxford Rd, Manchester, M13 9WL UK; 68 Maastricht University, Minderbroedersberg 4-6, 6211 LKZ Maastricht, Netherlands; 69 Medical University of Vienna, Spitalgasse 23, 1090 Wien, Austria; 70 Royal Devon & Exeter NHS Foundation Trust, Barrack Road, Exeter, Devon EX2 5DW UK; 71 Haematology Department, Royal Victoria Infirmary, Queen Victoria Rd, Newcastle upon Tyne, NE1 4LP UK; 72 The Katharine Dormandy Haemophilia Centre and Thrombosis Unit, Royal Free London NHS Foundation Trust, Pond St, Hampstead London, NW3 2QG UK; 73 San Matteo, Pavia, Viale Camillo Golgi, 19, 27100 Pavia, PV Italy; 74 Birmingham University NHS Foundation Trust, Level 1, Queen Elizabeth Hospital Birmingham, Mindelsohn Way, Edgbaston Birmingham, B15 2GW UK; 75 Queen Mary University of London, Mile End Rd, London, E1 4CS UK; 76 University Hospital Wales, Cardiff and Vale UHB Headquarters, University Hospital of Wales (UHW), Heath Park, Cardiff, CF14 4XW UK; 77 Department of Haematology, Great Ormond Street Hospital for Children NHS Trust, Great Ormond Street, London, WC1N 3JH UK; 78 Madsen Health Center, 555 Foothill Dr, Salt Lake City, UT 84112 USA; 79 Leicester Royal Infirmary, Infirmary Square, Leicester, LE1 5WW UK; 80 Department of Pediatrics, Perelman School of Medicine at the University of Pennsylvania, 34th Street & Civic Center Boulevard, Philadelphia, PA 19104 USA; 81 Division of Hematology, Children’s Hospital of Philadelphia, 3401 Civic Center Blvd, Philadelphia, PA 19104 USA; 82 Royal Hallamshire NHS Foundation Trust, Glossop Road, Sheffield, S10 2JF UK; 83 Maastricht University Medical Centre, Postbus, 5800, 6202 AZ Maastricht, Netherlands; 84 King’s College Hospital NHS foundation trust, Denmark Hill, Brixton London, SE5 9RS UK; 85 Gartnavel General Hospital, NHS Greater Glasgow and Clyde, 1055 Great Western Rd, Glasgow, G12 0XH UK; 86 Royal Brompton Hospital, Sydney St, Chelsea London, SW3 6NP UK; 87 University of Perugia, Piazza dell’Università, 06123 Perugia, PG Italy; 88 Institut Hospitalo-Universitaire LIRYC, PTIB, Hopital Xavier Arnozan, Pessac, Avenue du Haut Lévêque, 33604 Pessac, France; 89 Frimley Park Hospital, Portsmouth Rd, Frimley Camberley, GU16 7UJ UK; 90 NHS Blood and Transplant, Manchester Blood Centre, Plymouth Grove Manchester, M13 9LL UK; 91 Hull & East Yorkshire Hospitals NHS Trust, Anlaby Rd, Hull, HU3 2JZ UK; 92 Arthur Bloom Haemophilia Centre, University Hospital of Wales Heath Park, Cardiff, Wales, Heath Park Way Cardiff, CF14 4XW UK; 93 Glasgow Royal Infirmary, NHS Greater Glasgow and Clyde, 84 Castle St, Glasgow, G4 0SF UK; 94 University of Lincoln, Brayford Pool, Lincoln, LN6 7TS UK; 95 Southampton General Hospital, University Hospital Southampton NHS Foundation Trust, Tremona Road, Southampton Hampshire, SO16 6YD UK; 96 Sheffield Teaching Hospitals, Herries Road, Sheffield, S5 7AU UK; 97 Haematological Laboratory, Trousseau Children’s Hospital, 26 Avenue du Dr Arnold Netter, 75012 Paris, France; 98 Sandwell and West Birmingham Hospitals, Dudley Road, Birmingham, West Midlands B18 7QH UK; 99 Norfolk & Norwich University Hospital, Colney Ln, Norwich, NR4 7UY UK; 100 University Hospitals of North Midlands, Royal Stoke University Hospital, Newcastle Road, Stoke-on-Trent, ST4 6QG UK; 101 Haemophilia, Haemostasis and Thrombosis Centre, Hampshire Hospitals NHS Foundation Trust, Aldermaston Rd, Basingstoke, RG24 9NA UK; 102 Hadassah-Hebrew University Hospital, Jerusalem, 91120 Israel; 103 Department of Haematology, Guys and St Thomas’ NHS Foundation Trust, Guy’s Hospital, Great Maze Pond, London, SE1 9RT UK; 104 Emma Children’s Hospital AMC, Meibergdreef 9, 1105 AZ Amsterdam-Zuidoost, Netherlands; 105 Salisbury Hospital, Salisbury NHS Foundation Trust, Odstock Rd, Salisbury, SP2 8BJ UK; 106 University of Edinburgh, Old College, South Bridge, Edinburgh, EH8 9YL UK; 107 Pathology and Laboratory Medicine, University of Western Australia, Crawley, Western Australia, 35 Stirling Hwy, Crawley, WA 6009 Australia; 108 Wellcome Trust Sanger Institute, Wellcome Trust Genome Campus, Hinxton, CB10 1SA UK

Correction to: *Scientific Reports* 10.1038/s41598-017-14403-y, published online 22 January 2018

The original version of this Article contained a typographical error in the spelling of the consortium member Patrick Yu-Wai-Man which was incorrectly given as Patrick Yu Wai Man.

In addition, a supplementary file containing additional algorithms and analysis was omitted from the original version of this Article.

These errors have now been corrected in the HTML and PDF versions of the Article.

