# Supplementary material for: Telomerecat: A ploidy-agnostic method for estimating telomere length from whole genome sequencing data
Source: Sci Rep. 2018 Jan 22;8:1300. doi: 10.1038/s41598-017-14403-y (PMC5778012; doi:10.1038/s41598-017-14403-y)
Supplement: Supplementary file 1 — telomerecat-supplementary-information-file [file 41598_2017_14403_MOESM1_ESM.pdf]

Supplementary Information File:

Telomerecat: A ploidy-agnostic method for estimating telomere length from whole genome sequencing data

James HR Farmery[1,*], Mike L Smith[2], NIHR BioResource - Rare Diseases[3], and Andy G Lynch[1,4]

[1]Cancer Research UK Cambridge Institute, University of Cambridge, Li Ka Shing Centre, Robinson Way, Cambridge CB2 0RE, UK
[2]European Molecular Biology Laboratory (EMBL), Genome Biology Unit, 69117 Heidelberg, Germany.
[3]NIHR BioResource - Rare Diseases, Cambridge University Hospitals, Cambridge Biomedical Campus, Cambridge, CB2 0QQ, UK
[4]School of Mathematics and Statistics/School of Medicine, University of St Andrews, St Andrews, Fife, KY16 9SS, UK
[*]Correspondance: henry.farmery@cruk.cam.ac.uk

ABSTRACT

A companion Supplementary Information file for the paper listed above.

1 Algorithms

In this file we detail Algorithms that are pertinent to the method. Please see the algorithms placed through out this document.

2 Further MSC passage analysis

We see that TelSeq fails to identify the expected pattern of telomere shortening in the MSC passage samples (Figure 2, Main Text). We propose that the reason for this is a disconnect between telomere coverage and coverage at regions of the genome which have the same GC content.

The TelSeq method works by adjusting the amount of reads which contain a certain amount of the telomere hexamer, based on the number of non-telomere reads with 49%-51% GC content. The underlying assumption being that reads with similar GC contents are sequenced at the same rate.

We see in Figure 1 that when we plot all reads in the BAM file that contain more than 6 hexamers, as identified by TelSeq, the expected patterns are reproduced perfectly. However, the amount of reads with more than 6 hexamers must be normalised by some factor to account for sequencing depth. In the TelSeq algorithm this factor is the number of non-telomere reads with 49%-51% GC content. We see in Figure 2 how many of these reads reside in each sample. Clearly, SRR1020606 and SRR1022350 have many more of these reads than the other samples. When this normalising factor is applied to the read counts shown in Figure 1 the expected pattern is obscured.

We see then that the relationship between telomere reads and reads with the same GC is not consistent across all samples. This inconsistency causes TelSeq to mischaracterise the expected telomere length patterns. It would seem that Telomerecat's approach of estimating telomere coverage by boundary reads is a fairer reflection of coverage over telomere for these samples.

3 Testing Telomerecat using simulated data

To test the underlying principles of our method we applied Telomerecat to a set of simulated data. The simulation approach that we developed allowed us to generate reads from a sample with known telomere length. We could then compare the length estimates provided by Telomerecat with the underlying true telomere length. The simulation approach also allowed us to test

Algorithm 1 Algorithms to reduce noise on the error profile mask matrix E. Where $P_o = P_{max} - P_{min}$

```
function MASKCORRECTION(E)

    N ← NEIGHBOURCOUNT(E)
    C ← CONTINUOUSCOUNT(E)
    C^T ← CONTINUOUSCOUNT(T(E))
    E' ← An L × (P_o) matrix where E'_ij = 0
    for i in{0,1,...,P_o} do
        for j in{0,1,...,L} do
            if N_ij ≥ 4 or C_ij ≥ 4 or C^T_ij ≥ 4 then
                E'_ij = 1
            else
                E'_ij = 0
    return E'

function NEIGHBOURCOUNT(E)
    N ← An L × (P_max − P_min) matrix where N_ij = 0
    for i in{0,1,...,P_o} do
        for j in{0,1,...,L} do
            N_ij ← ∑_{x=−1}^{1} ∑_{y=−1}^{1} E_{i+x,j+y}
            N_ij ← N_ij − E_ij
    return N

function CONTINUOUSCOUNT(E)
    C ← An L × (P_o) matrix where C_ij = 0
    for i in{0,1,...,P_o} do
        start ← 0; count ← 0;
        for j in{0,1,...,L} do
            if E_ij == 1 then
                count ← count + 1
            else if E_ij == 0 then
                C_{i,start:j} ← count
                start ← j + 1;count ← 0
    return C
```

whether Telomerecat was influenced by the number of chromosomes in the sample and whether our method of interstitial filtering was well founded.

Pseudocode for the validation simulation is given in Algorithm 4. In essence, we use the ART simulator to simulate a set of reads from a hypothetical reference genome comprised of telomere, subtelomere and random DNA sequence. A diagram and explanation for the method used to create the hypothetical sequence is shown in Figure 3. The set of parameters used for this investigation are shown in 1. For the purposes of this investigation we generated estimations for 5000 samples with varying telomere length, subtelomere length, coverage and ploidy.

The results of this investigation show strong agreement between estimated telomere length and the true underlying telomere length (Figure 4A). We also confirmed that estimated telomere length did not appear to be biased by the number of chromosomes present in the simulated sequence (Figure 4B).

We were also reassured to find that extreme outliers were only seen in samples with low depth of sequencing and few chromosomes (Figure 4C).

The simulation approach detailed in this section is extremely limited in the extent to which it can demonstrate Telomerecat's ability to estimate telomere length accurately. It is not clear that our hypothetical genome model is a good likeness of a real chromosome. Particularly the interface between telomere and subtelomere. Furthermore, we suspect that the sequencing reads produced by the ART simulator contain less sequencing error in the telomere reads than actual sequencing experiments. As such this does not represent a validation of Telomerecat's ability to differentiate telomere reads suffering sequencing error and subtelomere reads.

Algorithm 2 Final step in producing the error profile

```
function InclusiveMask(E)

    maxIndicies ← an empty list
    for j in{0,1,...,L} do
        rowMaxima ← 0
        for i in{0,1,...,P_o} do
            if E_ij == 1 then
                rowMaxima ← j
        maxIndicies append rowMaxima
    E' ← An L × P_o matrix where E'_ij = 0
    for j in{0,1,...,L} do
        for i in{0,1,...,P_o} do
            if i ≤ maxIndicies[j] then
                E'_ij ← 1
    return E'
```

Algorithm 3 Sort read pairs into the Telomerecat the read types shown in Figure 9 (main text). We assume that the variables z, λ and L were calculated previously for each of the reads.

```
function getReadType(read1, read2)
    if  isTelomere(read1) and isTelomere(read2) then
        ▷ Both reads in the pair are telomere
        return F1
    else if isTelomere(read1) or isTelomere(read2) then
        ▷ Exactly one of the reads in the pair is complete
        teloRead ← read1 if isTelomere(read1) else read2
        if CCCTAA in teloRead.seq then
            return F2
        else
            return F4
    else
        ▷ Neither read is complete
        return F3

function isTelomere(read)
    z ← z_read
    λ ← λ_read
    L ← L_read
    if z < 1/10 · L then
        return TRUE
    else if E_{λ,z} == 1 then
        return TRUE
    else
        return FALSE
```

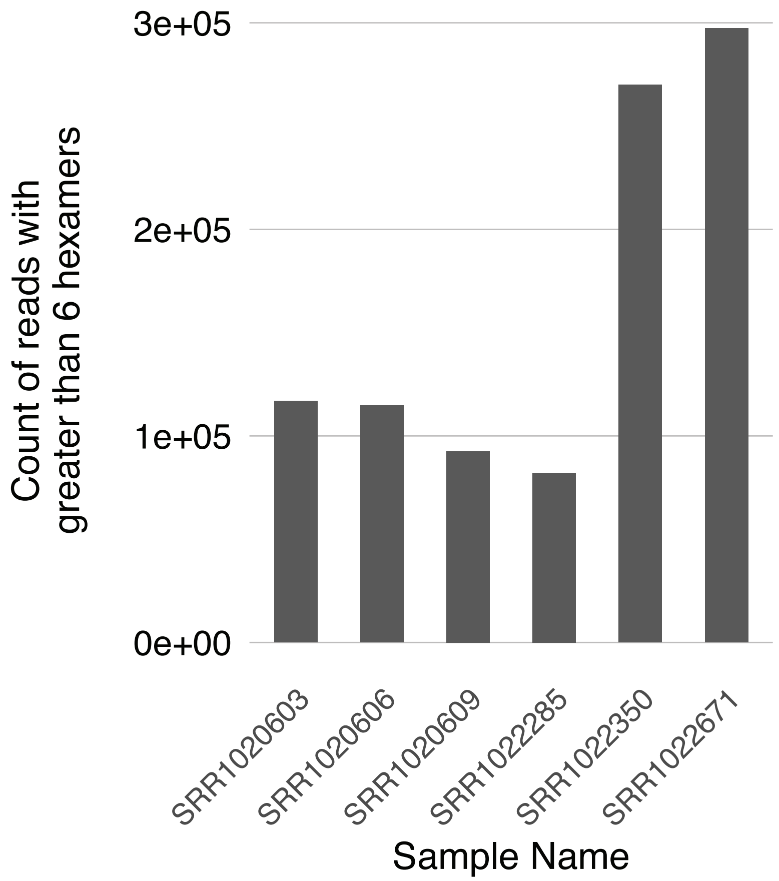

Figure 1. *Number of reads that have more than 6 occurrences of the telomere hexamer for each sample in the MSC passage experiment, as identified by TelSeq*

Despite the drawbacks of this approach it is still a useful investigation. The results lead us to believe that our method for inferring telomere coverage by observing the boundary between telomere and subtelomere is sound and yields to estimates unbiased by ploidy. We also see that the method identifies interstitial reads as per our expectation. It is clear that there is routinely a surplus of F2 reads in comparison to F4. The only reasonable explanation for this observation is that F2 and F4 reads are produced evenly at the site of the ITR however *only* F2 reads exist at the boundary between telomere and subtelomere.

This investigation shows us that our interpretation of telomere biology and how it is represented in WGS samples is sound. Using this high quality simulation data, Telomerecat is able to estimate telomere length to a high degree of accuracy.

4 Software information

All analysis completed using Telomerecat v3.1.1 (available as a release on github). Default settings were used for all analyses.

Where Telseq was used, the most recent version (v0.0.1) was used with default settings.

Algorithm 4 Estimate telomere length from an artificial DNA sequence. The parameters used for the investigation described in the text are given in Table 1. A diagram of the genome as generated by the GetGenome function is given in Figure 3
.

function VALIDATIONSIM(μ^t, σ^t, μ^s, σ^s, μ^c, σ^c, μ^d, σ^d)
  ▷ Draw from the normal distribution to decide the
  ▷ length of telomere (t), length of subtelomere (s),
  ▷ number of chromsomes (c) and depth of sequencing (d)
  $t \leftarrow \mathcal{N}(\mu^t, \sigma^t)$
  $s \leftarrow \mathcal{N}(\mu^s, \sigma^s)$
  $c \leftarrow round(\mathcal{N}(\mu^c, \sigma^c))$
  $d \leftarrow round(\mathcal{N}(\mu^d, \sigma^d))$

  ▷ Use number of t, s, and c to generate a random
  ▷ DNA sequence with interstitial telomere sequence
  $seq \leftarrow$ GETGENOME(t, s, c)

  ▷ Simulate reads from the hypothetical reference
  ▷ sequence using the ART simulator
  $bam \leftarrow$ SIMULATEREADS(seq, d)

  ▷ Estimate telomere length using Telomerecat
  $e \leftarrow$ ESTIMATETELOMERE(bam)

  ▷ Return the estimated and actual telomere length
  return e, t

Name	Value
μ^t	5
σ^t	2.5
μ^s	1.5
σ^s	1
μ^c	23
σ^c	10
μ^d	25
σ^d	20
Read length	100
Insert size	350

Table 1. *Parameters used in the validation simulation investigation*

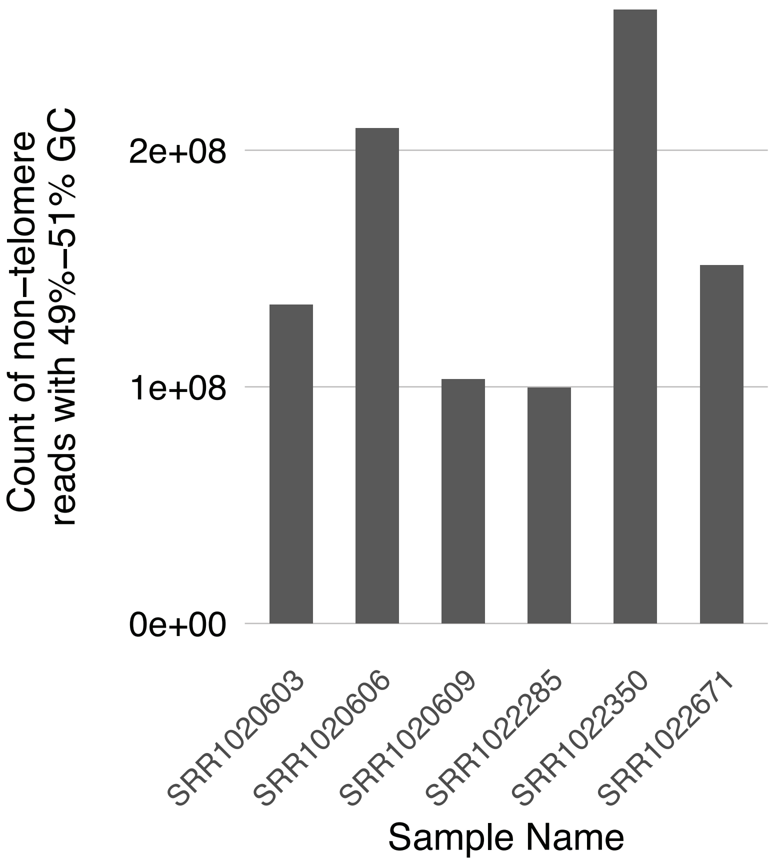

Figure 2. *Number of reads where the GC content is between 49% - 51% for each sample in the MSC passage experiment as identified by telomerecat*

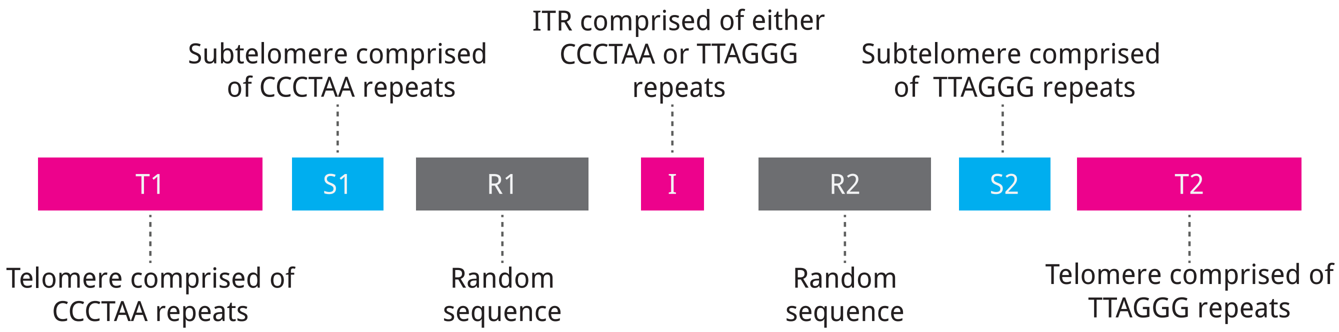

Figure 3. *A structural overview of the hypothetical sequence used for the validation simulation.* ***T1 & T2:****representation of telomere. These sections are comprised of the canonic telomere sequence repeated.* ***S1 & S2:*** *representation of subtelomere. This is simply the telomere sequence where ~10% of bases were mutated at random. Accordingly it bares a strong resemblance to telomere.* ***R1 & R2:*** *random DNA sequence in which the letters T,A,C,G appear uniformly throughout.* ***ITR:*** *A stretch of telomere repeats. A coin is flipped to decide whether the sequence will appear as CCCTAA or TTAGGG in the reference. The lengths of the subtelomere and telomere sections of the reference are sampled from the normal distribution as per Algorithm 4. The R1 and R2 sections are always 1.5KB long and the ITR always constitute 17 instances of the relevant canonic sequence (102bp).*

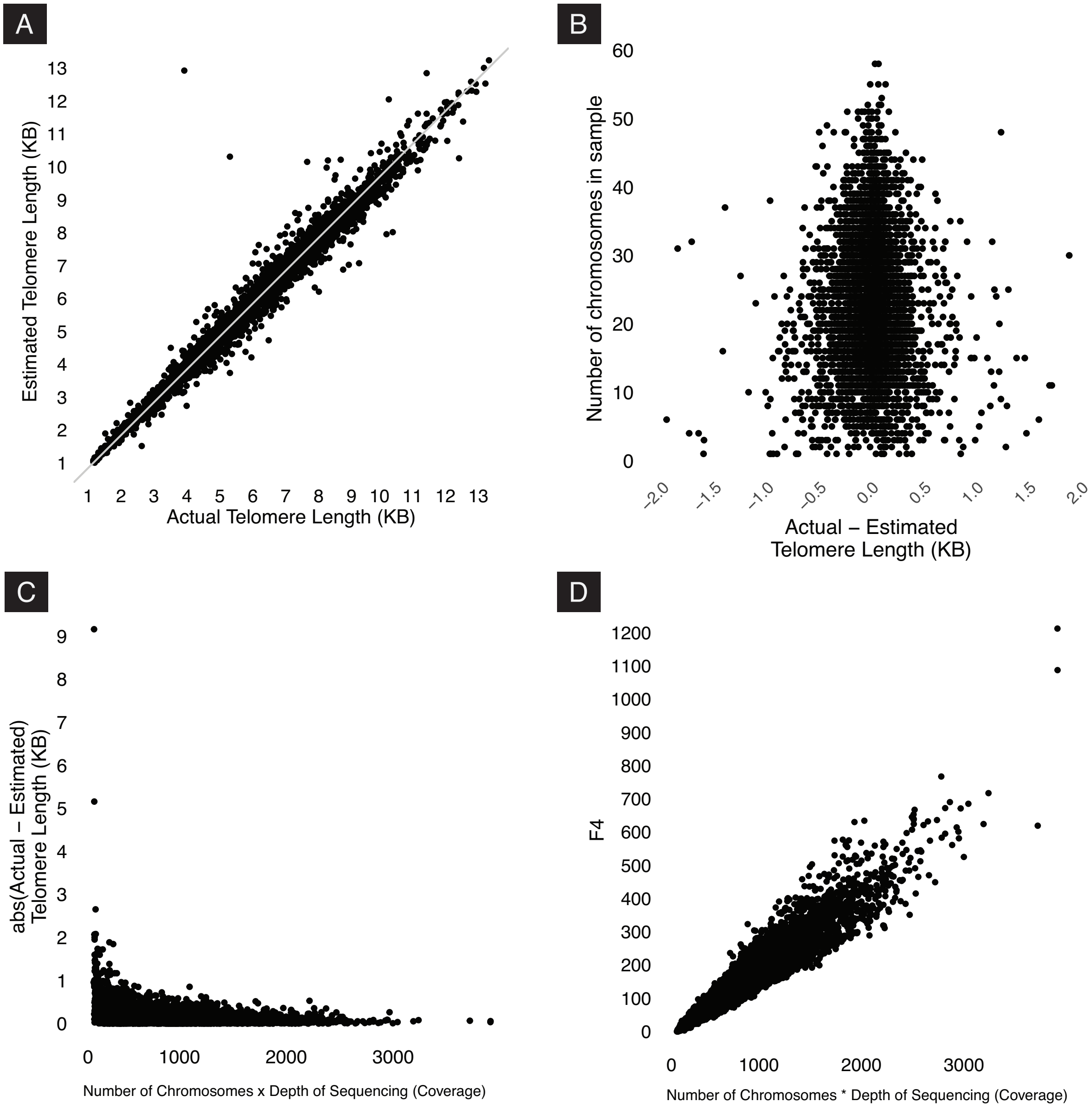

Figure 4. *Results of the validation simulation **(A):** A plot of estimated vs actual telomere length. Light gray line is line of agreement. It appears that estimated and actual telomere length strongly agree **(B):** Difference between estimated and actual telomere length plotted by number of chromosomes in the sample. We see that the difference between estimates is centered around 0 regardless of chromosome count. Extreme outliers have been cropped from this plot. **(C):** Number of chromosomes multiplied by depth of sequencing (a proxy for reads in the sample) plotted against absolute difference between estimated and actual telomere length. We see that samples with the poorest estimation were produced by samples with low sequencing coverage and chromosome count **(D):** F4 vs Number of chromosomes multiplied by depth of sequencing*
